# Association of naturally acquired type-specific HPV antibodies and subsequent HPV re-detection: systematic review and meta-analysis

**DOI:** 10.1186/s13027-023-00546-3

**Published:** 2023-11-08

**Authors:** Kana Yokoji, Katia Giguère, Talía Malagón, Minttu M. Rönn, Philippe Mayaud, Helen Kelly, Sinead Delany-Moretlwe, Mélanie Drolet, Marc Brisson, Marie-Claude Boily, Mathieu Maheu-Giroux

**Affiliations:** 1 Department of Epidemiology and Biostatistics, School of Population and Global Health, McGill University, 2001 Avenue McGill College, Suite 1200, Montreal, QC H3A 1G1 Canada; 2 Institut national de santé publique du Québec, Quebec City, Canada; 3 Division of Cancer Epidemiology, Gerald Bronfman Department of Oncology, McGill University, Montreal, Canada; 4 Department of Global Health and Population, Harvard T.H. Chan School of Public Health, Boston, USA; 5 Department of Clinical Research, London School of Hygiene and Tropical Medicine, London, UK; 6 Wits RHI, University of Witwatersrand, Johannesburg, South Africa; 7 Centre de recherche du CHU de Québec - Université Laval, Quebec City, Canada; 8 MRC Center for Global Infectious Disease Analysis, School of Public Health, Imperial College London, London, UK

**Keywords:** Human papillomavirus, HIV/AIDS, Cervical cancer, Natural immunity, Infection, Meta-analysis

## Abstract

**Background:**

Understanding the role of naturally acquired (i.e., infection-induced) human papillomavirus (HPV) antibodies against reinfection is important given the high incidence of this sexually transmitted infection. However, the protective effect of naturally acquired antibodies in terms of the level of protection, duration, and differential effect by sex remains incompletely understood. We conducted a systematic review and a meta-analysis to (1) strengthen the evidence on the association between HPV antibodies acquired through past infection and subsequent type-specific HPV detection, (2) investigate the potential influence of type-specific HPV antibody levels, and (3) assess differential effects by HIV status.

**Methods:**

We searched Embase and Medline databases to identify studies which prospectively assessed the risk of type-specific HPV detection by baseline homologous HPV serostatus among unvaccinated individuals. Random-effect models were used to pool the measures of association of naturally acquired HPV antibodies against subsequent incident detection and persistent HPV positivity. Sources of heterogeneity for each type were assessed through subgroup analyses stratified by sex, anatomical site of infection, male sexual orientation, age group, and length of follow-up period. Evidence of a dose-response relationship of the association between levels of baseline HPV antibodies and type-specific HPV detection was assessed. Finally, we pooled estimates from publications reporting associations between HPV serostatus and type-specific HPV detection by baseline HIV status.

**Results:**

We identified 26 publications (16 independent studies, with 62,363 participants) reporting associations between baseline HPV serostatus and incident HPV detection, mainly for HPV-16 and HPV-18, the most detected HPV type. We found evidence of protective effects of baseline HPV seropositivity and subsequent detection of HPV DNA (0.70, 95% CI 0.61–0.80, N_E_ = 11) and persistent HPV positivity (0.65, 95% CI 0.42–1.01, N_E_ = 5) mainly for HPV-16 among females, but not among males, nor for HPV-18. Estimates from 8 studies suggested a negative dose–response relationship between HPV antibody level and subsequent detection among females. Finally, we did not observe any differential effect by baseline HIV status due to the limited number of studies available.

**Conclusion:**

We did not find evidence that naturally acquired HPV antibodies protect against subsequent HPV positivity in males and provide only modest protection among females for HPV-16. One potential limitation to the interpretation of these findings is potential misclassification biases due to different causes.

**Supplementary Information:**

The online version contains supplementary material available at 10.1186/s13027-023-00546-3.

## Introduction

Human papillomavirus (HPV) is one of the most common sexually transmitted viruses, affecting 50% of sexually active individuals at least once in their lifetime [1]. Infection with HPV is associated with 7–8% of all human malignancies and accountable for 96% of cervical cancer, 93% of anal cancers, 64% of vaginal cancers, 51% of vulvar cancer, 36% of penile cancers, and 63% of oropharyngeal carcinomas [2]. Among all high-risk HPV oncogenic types (HR-HPV), HPV-16 and HPV-18 account for 60–80% of all cervical cancers, one of the most commonly diagnosed cancers and a leading cause of cancer death in women in many low-income countries [3–7].

Most HPV infections clear within 1–2 year by cell-mediated immune response and generation of serum neutralizing antibodies (IgG) against the capsid L1 protein of HPV [1, 8]. However, while most of infections are cleared, some persist for years, which can lead to cell abnormalities and potentially to cancer, if not promptly diagnosed and adequately treated [9]. Studies have shown the potential protective effect of neutralizing antibodies against subsequent infections when the immune response is initiated by HPV vaccines [10]. Currently, several prophylactic vaccines are available, which include: bivalent vaccines that offer protection against HPV-16/18, quadrivalent vaccines against HPV-6/11/16/18, and nonavalent vaccines against HPV-6/11/16/18/31/33/45/52/58 [11]. The high protection (> 90%) against vaccine-targeted HPV-type incident infections, and HPV-related abnormalities and precancerous lesions, have been demonstrated by clinical studies [10, 12]. However, the extent to which naturally acquired, or infection-induced, antibodies can help prevent HPV reinfection remains poorly understood, especially with respect to the level and the duration of protection. One among many challenges in investigating the effect of naturally acquired antibodies against subsequent HPV infection is the role of latency and deposition, and consequently, the difficulties around determining whether the incident HPV DNA detection truly represents true re-infection, or rather a deposition from sexual partners and/or from cross-site contamination, or reactivation of the virus [13].

The evidence base regarding risk of subsequent infections with HR-HPV is mixed. Several studies found no effect of naturally-acquired antibodies in reducing the risk of subsequent HPV detection in men [14–17] and in women [18–20] while others found some levels of protection among women [21–28]. These mixed findings could be attributed to varying study designs, heterogeneous study populations, and different laboratory protocols used to confirm HPV detection, while small sample sizes might have increased the uncertainty of estimates. The most recent meta-analysis of naturally acquired HPV antibodies against subsequent genital HPV infection, published in 2016, has estimated a 30–35% reduction in the risk of subsequent HPV-16/18 genital infection among women, but not among men [29]. However, the study was not able to explore sources of heterogeneity aside from sex and it did not assess the influence of HPV antibody levels or HIV status on type-specific re-infection rates.

Current evidence points to multiple interactions between HIV and HPV infections, due to similar risk factors, and biological and immunological factors [30]. Studies have suggested that the risk of acquiring HPV infection, persistent infection, and disease progression is increased among people living with HIV (PLHIV). However, the impact of HIV infection on the natural immune response to HPV following natural infection is less understood [31, 32]. Acquiring quantitative estimates of the risk of subsequent HPV infection among PLHIV is particularly important for assessing the impact of HPV vaccination programs in settings where HIV prevalence is highest and informing parameterization of mathematical models to understand the epidemiology of HPV infection and cervical cancer in high-risk populations.

The objectives of this study were threefold. First, we updated the existing evidence on the association between naturally acquired immunity and subsequent type-specific HPV detection through a systematic review and meta-analysis. We also performed several subgroup analyses to further investigate sources of heterogeneity in the association between natural immunity and risk of subsequent HPV detection (stratified by anatomic site of detection, sex, sexual orientation, age group). Second, we investigated the influence of HPV antibody levels to type-specific HPV detection risk. Third, we examined if HPV detection risk differ by HIV status. We performed analyses on all HPV types where possible but reported results for HPV-16 and HPV-18 in the main results.

## Methods

### Search strategy and selection criteria

All results of this study are reported according to the PRISMA guidelines (Additional file 1: Table S1). We searched the Embase and MEDLINE databases for articles in English, French, and Japanese, published up to May 2022. We used key search terms related to four domains—HPV, study design, antibodies, and viral DNA—to capture prospective studies assessing an association between HPV serostatus at baseline (history of previous HPV infection) and subsequent type-specific HPV detection among HPV-unvaccinated individuals (HPV DNA detection at follow-up among DNA-negative participants at baseline; see Additional file 1: Table S2).

Two reviewers (KY and KG) independently screened titles and abstracts of potentially eligible articles after removing duplicate records and resolving any discrepancies in the selection. Full texts of all potentially relevant articles were then screened. We excluded publications that did not report estimates of association between baseline HPV serostatus and type-specific HPV incident detection at follow-up, or that did not provide sufficient data to derive them. Finally, the reference lists of all included publications were screened to identify additional relevant studies [29].

### Data extraction

Data were retrieved by two reviewers (KY and KG) and any discrepancies were resolved by consensus. We extracted the reported crude and adjusted estimates, or the data to derive it, of any measures of association between type-specific serostatus of any reported HPV-type and incident detection of HPV DNA at follow-ups. These include the incidence rate ratio (IRR), cumulative incidence ratio or risk ratio (RR), hazards ratio (HR), or the odds ratio (OR) and their 95% confidence intervals (95%CI) or the data to derive unadjusted RR or IRR (herein referred to as *self-calculated*) from reported counts and/or incidence rates (Additional file 1: Text 2). We also extracted information on participants characteristics and HPV detection (i.e., sex, age, population type, HIV status, HPV type, infection site), study characteristics (i.e., country, study design, sample size, follow-up duration), and quality indicators (e.g., type of tests used for HPV serology and HPV DNA detection, variables adjusted for). For the third objective, we also extracted information on HIV disease stage, ART or other treatment status, and CD4+ cell counts of individuals living with HIV where possible.

We calculated the pooled measure of association (on the relative risk scale) and 95%CI using inverse variance weights and the DerSimonian-Laird random-effect method on the logarithmic scale (results are then presented on the original scale) [33]. To maximize the number of estimates, we pooled all measures of association (IRR, RR, HR, OR) in our main meta-analysis but assessed their influence in subgroup analyses. The I^2^ statistic was used to assess heterogeneity between study estimates [34]. To avoid including duplicated estimates on same participants, we only selected one estimate from studies reporting on the same study population (e.g., HIM cohort study) for each HPV type and infection site. Finally, estimates that were adjusted for potential confounders were chosen over unadjusted estimates, if available, for the pooled analysis. However, we also compared crude and adjusted estimates in a sensitivity analysis described below.

For the first objective, we used two different outcomes: (1) incident type-specific HPV detection, defined as the first detection of homologous HPV DNA at follow-up among HPV DNA-negative individuals at baseline (as defined by included studies), and (2) persistent HPV positivity, defined as the detection of homologous HPV DNA at two or more consecutive follow-up visits within a 6- or 12-month interval among HPV DNA-negative individuals at baseline. For both outcomes, we focused on HPV-16 and HPV-18 in our main meta-analyses, since these HPV types were the most commonly reported [35]. We presented results on other HPV types for the first outcome in Additional file 1 for completion. For the outcome of incident HPV detection, we also performed subgroup analyses to investigate whether heterogeneity between study estimates could be explained by sex, HPV detection site (cervical/cervicovaginal, penile/scrotal, anal, oral), sexual orientation for male (men who have sex with men [MSM], men who exclusively have sex with women [MSW]), and age group (≤ 30, >30 years). Subgroup analyses were restricted to HPV-16 and HPV-18 only, based on the availability of extracted data from studies. Differences between subgroups were tested using the Q-test or subgroup differences (two-sided, 5% alpha level). We did not perform subgroup analyses for the outcome of persistent infection due to low number of estimates available for stratification. We also performed sensitivity analyses to assess study quality and potential biases due to heterogeneity in study designs (described in the next section).

To assess evidence of a dose–response relationship between baseline type-specific HPV antibodies and same type HPV detection, we plotted the measures of association by levels of HPV antibodies (i.e., terciles, quartiles, low versus high) from publication reporting this data for HPV-16 and HPV-18. We also performed meta regression using the estimates from the publications above as individual data point. Given heterogeneity in the categorization of antibody titres, we compared and pooled the risk of detection in the highest category (i.e., “high”, “highest quartile”, “highest tercile”) to the lowest one (i.e., “low”, “lowest quartile”, “lowest tercile”).

Finally, for the third objective, we pooled estimates reporting associations between HPV serostatus and type-specific HPV detection by baseline HIV status. Differences between HIV positive and HIV negative individuals were assessed using the Q statistic. Again, these analyses were restricted to HPV-16 and HPV-18 only, based on the availability of extracted data from studies.

### Sensitivity analyses

We tested the influence of each individual study estimate on the overall pooled estimates using leave-one-out sensitivity analyses. We also performed several sensitivity analyses to assess whether study estimates varied due to potential biases. Specifically, we compared (1) crude versus adjusted estimates; (2) reported versus estimates calculated from raw data reported in publications; (3) types of measures of associations (IRR, HR, RR, OR); (4) methods used for serotyping (neutralizing versus non-neutralizing assay); (5) primers set used for HPV-DNA detection (by decreasing test sensitivity: PGMY09/11, SPF10, MY09/11) [36]; and (6) lengths of follow-up periods. For the first sensitivity analysis, we used two different approaches: we compared crude versus adjusted estimates from all studies, as well as crude versus adjusted estimates in the subset of studies reporting both.

Study quality was assessed using a modified version of the *Newcastle–Ottawa-Scale* (NOS) for cohort studies (Additional file 1: Text 3) [37]. Scores were assigned on a continuous scale from 0 to 12 stars based on 8 criteria including the strength of study design for an inference, methods to assess the exposure/outcome, etc. Two independent reviewers assigned the scores independently, and any discrepancies were discussed to reach an agreement. We then categorized the studies into low, medium, and high quality for scores ranging from 0 to 4, 5 to 8, and 9 to 12, respectively. Finally, the risk of publication bias was further examined by funnel plots and Egger’s tests of symmetry [38]. We performed all analyses with R (version 4.0.3), using the “meta” package [39].

## Results

### Search results

**Fig. 1:**
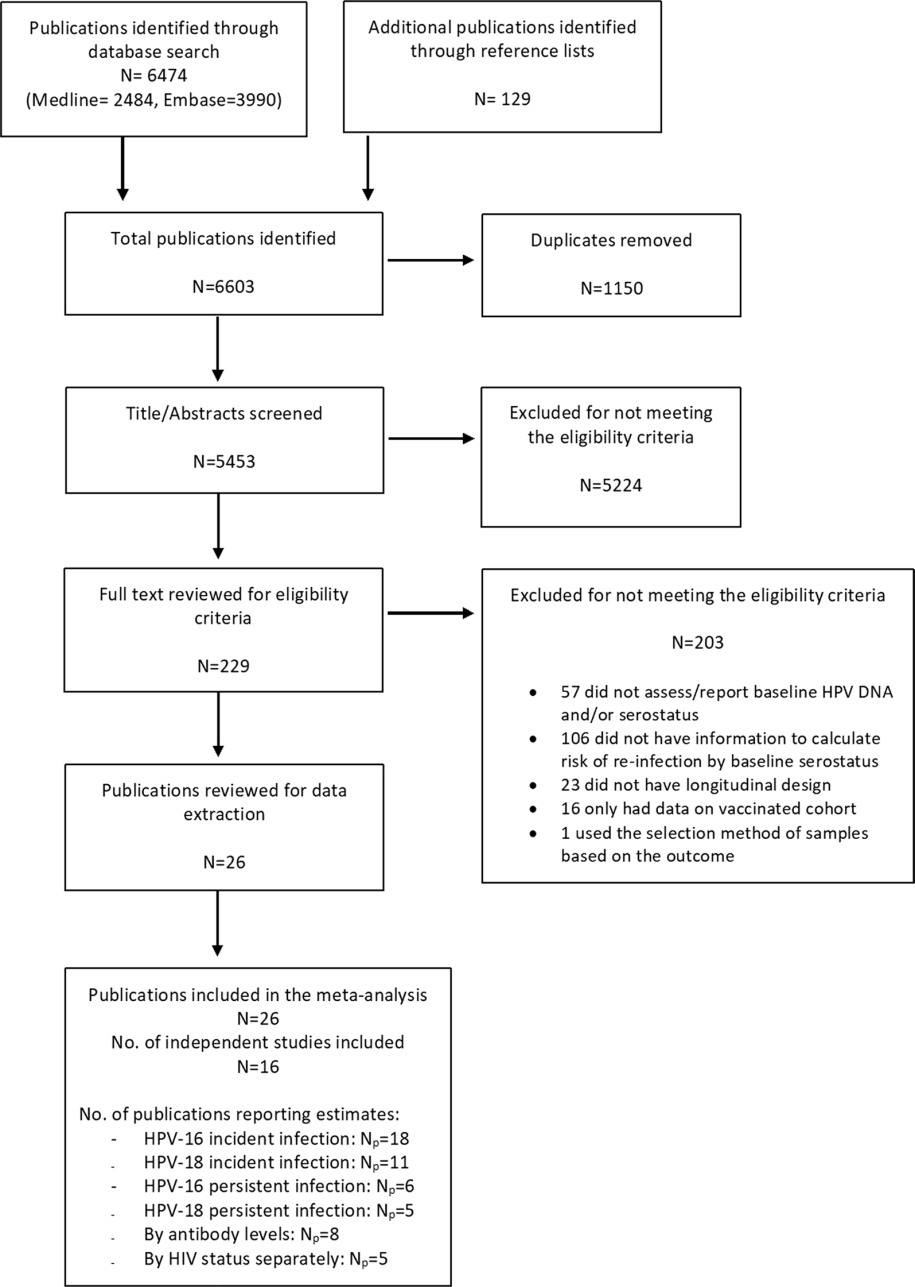
Flow diagram of the study selection for the systematic review and meta-analysis of the association between baseline HPV serostatus and type-specific HPV re-detection

The search strategy yielded 6474 records and an additional 129 publications were identified through screening of the reference lists of included publications (Fig. 1). After removal of duplicates, a total of 5453 publications were assessed for eligibility through title and abstract screening, of which 45,224 were excluded for non-relevance. In total, 26 publications reporting on 16 independent studies (n = 62,363 participants) met our eligibility criteria and were included in our study, which adds 12 more publications to the most recent meta-analysis. These publications reported a total of 92 different estimates of the associations between HPV serostatus at baseline and incident type-specific HPV detection for any HPV types reported.

### Study characteristics

Of the 16 included independent studies, 9 were prospective observational cohort studies and 7 were randomized clinical trials on HPV vaccines (Table 1). HPV-16 (number of publications [N_p_] = 26) and HPV-18 (N_p_ = 14) were the two most common HR-HPV types documented in these studies (for other types, see Table 1). Eighteen publications reported estimates of incident detections and 4 publications on 6-month and 12-month persistent detections. Most publications (N_p_ = 19) focused on females and HPV detection at cervical or cervicovaginal sites, while 6 publications focused on males, and 1 publication included both sexes as participants. Most publications reported on multi-country studies but only one study included cohorts from sub-Saharan Africa (Burkina Faso, South Africa). The most common infection sites assessed among males were penile and/or scrotal sites (N_p_ = 4), followed by oral (N_p_ = 3) and anal (N_p_ = 2) sites. A total of 8 publications also provided estimates by baseline levels of HPV antibodies. One publication was restricted to HIV-positive females, and another four reported estimates stratified by HIV status among females or both sexes. SPF10 was the most common DNA primer set used for HPV DNA detection (N_p_ = 14), and most of the studies used non-neutralizing serologic assay for the detection of serum HPV antibodies (N_p_ = 24).

**Table 1:** Summary of study characteristics of publications included in the meta-analysis of the association between baseline HPV serostatus and type-specific HPV detection (N_p_ = 26)

References	Study^a^	Population^b^	Mean/median age^c^	N	Follow up duration^d^	HPV type	Serologic assay^e^	DNA assay primers	Detection site^f^	Outcome measure^g^	Effect measure^h^	Variables controlled for	Study quality
Wilson et al. [28]	ALTS (RCT)	Females	NA	2302	Dur = 24	16, 18, 31,33, 35, 45, 52, 58	Luminex	PGMY9/11	Cervical	Incident detection	OR	New sex partner during follow up	High
Eldridge et al. [50]	ALTS (RCT)	Females with ASCUS or LSIL	Never smoker: Mean = 28.9 (sd = 9.6), Former smoker: 35 (12), Current smoker: 27.5 (8.4)	1976	Dur = 24	16	Luminex	PGMY9/11	Cervical	Incident detection	aOR	Age, age at sexual debut, lifetime no. of sexual partners	Medium
S]afaeian et al. [25]	CVT (RCT)	Young females	Median = 21 IQR = 19–23	2813–2950	Dur = 48	16, 18	VLP-ELISA	SPF10	Cervical	Incident detection, Ab level in tertile	aIRR	Age, education, marital status, lifetime no. of partners, and smoking status	Medium
Lin et al. [22]	CVT (RCT)	Young females	NA	388	Max = 48	16	VLP-ELISA	SPF10	Cervical	Incident detection, Ab level in tertile	OR	None	Medium
Robbins et al. [51]	CVT (RCT)	Females	NA	488	Dur = 48	16, 18	Luminex	SPF10	Cervical	Incident detection	aOR	Lifetime no. of sexual partners at enrollement	Medium
Kelly et al. [40]	HARP (Cohort)	Females living with HIV	NA	604	Median = 16	16, 31, 33, 35, 52, 58, 18, 39, 45, 59, 68, 56, 6, 11, 73	Multiplex PsV-Luminex assay	SPF10	Cervical	Incident detection	aOR	Age, smoking, condom, vaginal washing, bacterial vaginosis, chlamydia trachomatis, trichomonas vaginalis, CD4 count, and antiretroviral therapy status at baseline, and seropositivity for HPV type from same family group	Medium
Viscidi et al. [20]	HERS (Cohort)	Females	NA	413	Max = 72	16, 18, 31, 35, 45	VLP-ELISA	MY09/11	Cervico-vaginal	Incident detection, HIV-/ +	aHR	Time-varying number of male sexual partners in the last 6 months at each study visit, and baseline CD4 (for HIV-positive women only)	Medium
Beachler et al. [14]	HIM (Cohort)	MSM	Mean = 33.1 (sd = 10.1)	475	Median = 50.4	16	VLP-ELISA	PGMY9/11	Penile, anal	Incident detection, Ab level in tertile	aHR	Age, country, sexual orientation, lifetime no.of sex partners, no. of recent sex partners, and circumcision status	High
Lu et al. [52]	HIM (Cohort)	Males	Mean = 29.8 (sd = 8.1)	285	Median = 15.5	16, 18	VLP-ELISA	PGMY9/11	Penil	Incident detection	IRR	None	Medium
Lu et al. [53]	HIM (Cohort)	Males	NA	2187	Median = 24	16	VLP-ELISA	PGMY9/12	Penil	Incident detection	RR/aHR	Age,sexual orientation, lifetime no. of sex partners, circumcision, no. of new sex partners	High
Pamnani et al. [16]	HIM (Cohort)	Males	NA	4103	Dur = 48	16, 18	VLP-ELISA	Not reported	Penil	Incident detection, persistent detection (6mo)	aHR	Marital status, alcohol use, lifetime male sexual partners, new female partners in past 6–12 months	Medium
Pierce Campbell et al. [17]	HIM (Cohort)	Males	Median = 32 IQR = 24–41	1618	Median = 12.7	6, 16	VLP-ELISA	PGMY9/12	Oral	Incident detection	HR	None	Medium
Mooij et al. [15]	H2M (Cohort)	MSM	NA	719	Dur = 12	16, 18	Luminex	SPF10	Anal	Incident detection, Ab level in tertile, HIV-/ +	aHR	HIV status, age, smoking, no.of lifetime male sex partners, no.of recent anal sex partners, anal sex position in the last 6 months, and having been fisted in the last 6 months	Medium
Herrero et al. [54]	NCT00128661 (RCT)	Young females	NA	2677	Median = 50.4	16, 18	VLP-ELISA	SPF10	Cervical	Persistent detection (6/12 mo)	IRR	None	Medium
Konno et al. [55]	NCT00929526 (RCT)	Young females	Mean = 22.4 (sd = 1.7)	752	Max = 48	16, 18	VLP-ELISA	SPF10	Cervical	Incident detection	RR	None	Medium
Yao et al. [56]	NCT01735006	Females	NA	3476	Dur = 24	16, 18	PBNA, VLP-ELISA	SPF10	Cervical	Incident detection	HR	Age at enrolment	High
Viscidi et al. [19]	NHS (Cohort)	Females	NA	7046	Dur = 60–84	16, 18, 31	VLP-ELISA	MY09/11	Cervical	Incident detection, Ab level in tertile	RR	None	Medium
Wentzensen et al. [27]	NHS (Cohort)	Females	NA	974	Dur = 84	16, 18, 6, 11	VLP-ELISA	PGMY9/11	Cervical	Incident detection	aOR	Stratified sampling	Medium
Beachler et al. [57]	POPS/MACS (Cohort)	MSM, females	NA	756	Max = 42	16, 33, 45	VLP-ELISA	PGMY9/11	Oral	Incident detection, Ab level in tertile, HIV-/ +	aOR	Age, gender, smoking status, no.of recent and lifetime oral sex partners, HIV/CD4 status, study site, and frequency of recent toothbrushing	Medium
Szarewski et al. [58]	PATRICIA (RCT)	Young females	NA	9325	Mean = 39.4	16, 18	VLP-ELISA	SPF10	Cervical	Incident detection, persistent detection (12 mo)	IRR (self calculated)	None	Medium
Castellsague et al. [21]	PATRICIA (RCT)	Young females	NA	8225	Max = 48	16, 18	VLP-ELISA	SPF10	Cervical	Incident detection, Ab level in quartile	aIRR	Marital status, tobacco exposure, age at first sexual intercourse, no. of recent sexual partners, no. of sexual partners (past year), pregnancy, sexually transmitted infection history, and region	Medium
Safaeian et al. [24]	PATRICIA/CVT	Young females	Mean = 20.4 (sd = 3)	9337 (PATRICIA), 3736 (CVT)	Dur = 48	16, 18	VLP-ELISA	SPF10	Cervical	Incident detection, Ab level in quartile	aIRR	Study, geographical region, marital status, cigarette smoking, lifetime no. of sexual partners, Chlamydia trachomatis infection at enrollment, age at first sexual intercourse, and previous pregnancy	Medium
Triglav et al. [26]	Slovenian HPV Prevalence Study (Cohort)	Females	Mean = 37.2	2199	Dur = 36	16	Multiplex PsV-Luminex assay	not reported	Cervical	Incident detection	OR	Age	Medium
Moscicki et al. [59]	University of California HPV natural history study (Cohort)	Females	Seropositive: Mean = 19.76 (2.14) Seronegative: 18.93 (2.06)	1543	Max = 168	16	Luminex	PGMY9/11	Cervical	Incident detection	IRR	None	Medium
Rosillon et al. [23]	VIVIANE (RCT)	Females	NA	2687–2705	Dur = 84	16, 18	VLP-ELISA	SPF10	Cervical	Incident detection, persistent detection (6/12 mo)	aHR	Region, age at inclusion, age at first sexual intercourse, marital status, smoking status at baseline, no.of sexual partners during the past year, previous pregnancy, history of Chlamydia trachomatis infection, history of HPV infection/treatment or nonintact cervix	Medium
Viscidi et al. [18]	WIHS (Cohort)	Females	Median = 36–45	2559	Max = 18	16	VLP-ELISA	MY09/11	Cervico-vaginal	Incident detection, HIV-/ +	HR	None	Medium

^a^POPS/MACS = the Persistent Oral human Papillomavirus Study (POPS) nested within the Multicenter AIDS Cohort Study (MACS) (Conducted in US); HIM = the Human Papillomavirus Infection in Men study (US, Brazil, Mexico); PATRICIA = the Papilloma Trial Against the cancer in Young Aduls (Australia, Belgium, Brazil, Canada, Finland, Germany, Italy, Mexico, Philippines, Spain, Taiwan, Thailand, UK, US); ALTS = the Atypical Squamous Cells of Undetermined Significance (ASCUS) Low-Grade Squamous Intraepithelial Lesion (LSIL) Triage Study (US); NCT001288661 (Costa Rica); HARP = HPV in Africa Research Partnership (South Africa); NCT00929526 (Japan); CVT = the Costa Rica HPV16/18 Vaccine Trial (Costa Rica); H2M = HIV& HPV in MSM (the Netherlands); University of California HPV natural history study (US); VIVIANE = Human Papillomavirus Vaccine Immunogenicity And Efficacy (Australia, Canada, Mexico, the Netherlands, Peru, Philippines, Portugal, Russia, Singapore, Thailand, the UK, and the USA); Slovenian HPV Prevalence Study (Slovenia); WIHS = the Women’s Interagency HIV (US); NHS = the Guanacaste Natural History Study; HERS = the HPV Epidemiology Research Study (Costa Rica). ^b^ MSM = Men who have sex with men; MSW = Men who have sex with women; MSWM = Men who have sex with women and men. ^c^ Mean or median age of the participants is reported (based on what is reported in the publication). IQR = interquartile range; sd = standard deviation. When only a range of age is reported as a median or mean, the age range is indicated. ^d^Dur = planned duration of follow up period. Max = Maximum follow up period. Median = Median follow up period. ^e^VLP-ELISA = Virus-Like Particle Enzyme-Linked Immunosorbent Assay; PsV-Luminex assay = Pseudovirion-based Luminex assay. ^f^Site of infection studied. "Penile" includes penile and scrotal site. ^g^Types of the outcome of interest reported in each publication. Ab level in tertile/ quartile = publications reporting estimates of association by HPV antibody level; HIV − / +  = publications reporting separate estimates by baseline HIV status. ^h^Effect measure reported as the primary outcome measure by the publication. OR = odds ratio; HR = Hazards ratio; RR = Risks ratio; IRR = Incidence rates ratio (“a” prefix for adjusted estimates)

Four publications were classified as having “high” study quality, and 22 as “medium” study quality, based on the criteria regarding the assessment of exposure, comparability, and outcome assessment (Additional file 1: Table S3). There was no single unique criterion that determined the “high” quality of studies; however, one of the key criteria was the proportion of participants lost to follow-up.

### Association between HPV serostatus and type-specific incident HPV detection

**Fig. 2:**
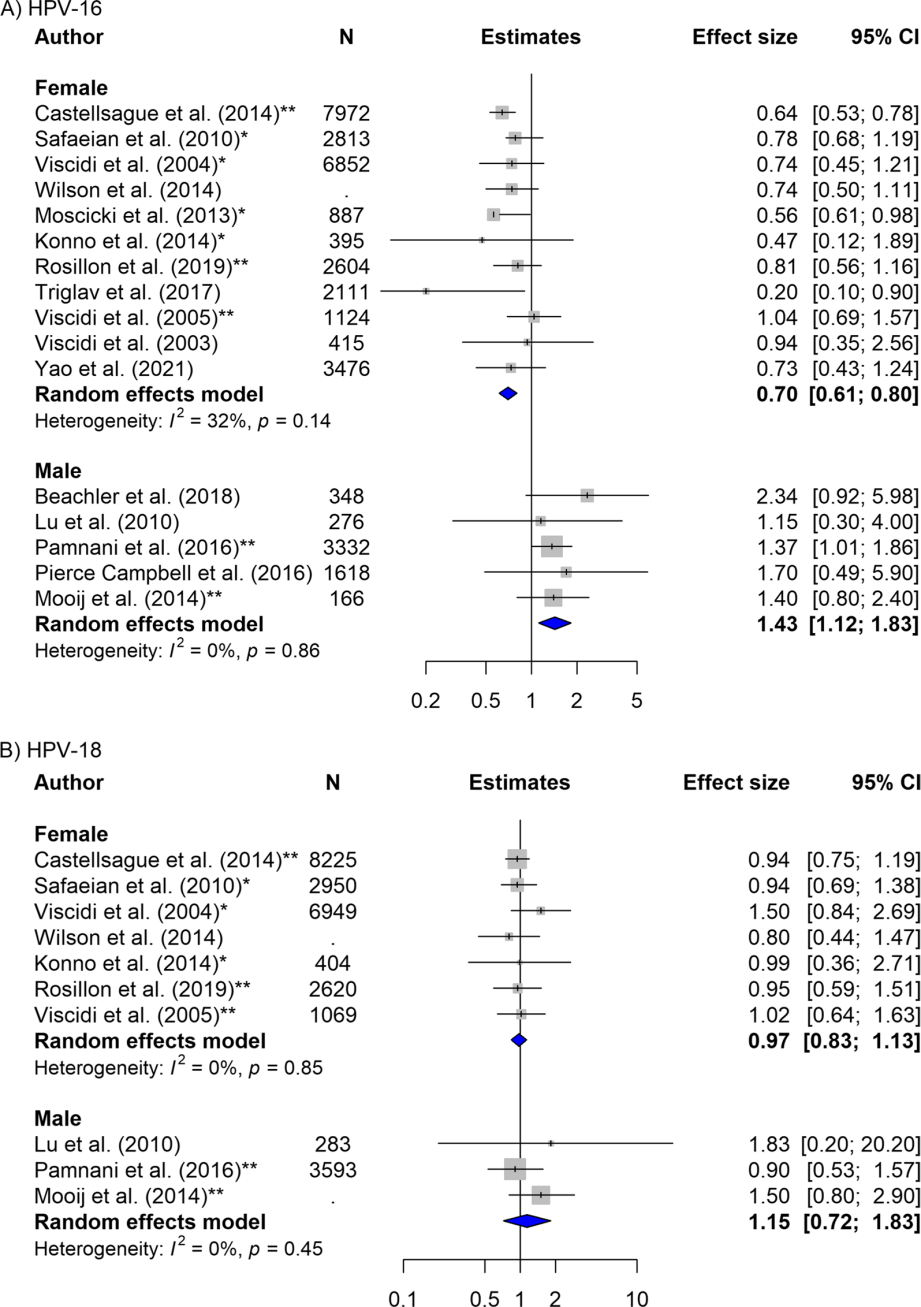
Forest plot of the association between baseline HPV serostatus and type-specific HPV incident detection by sex for **A** HPV-16, and **B** HPV-18. *Indicates estimates that are self-calculated using the provided data. **Indicates estimates that are adjusted for confounders

As the direction of association varied greatly by sex, the overall pooled estimates are difficult to interpret (Tables 2, 3) and we only presented overall estimates for completeness. For females, the risk of incident detection among baseline-HPV-16 seropositive was reduced compared to those baseline-seronegative females (0.70, 95% CI 0.61–0.80, N_E_ = 11). In reverse, for males, the risk of incident detection among baseline-HPV seropositive individuals was increased compared to those baseline-seronegative (HPV-16: 1.43, 95% CI 1.12–1.83, N_E_ = 5) (Table 2, Fig. 2). The results for HPV-18 were suggestive of similar direction of association but with wider confidence intervals (Females: 0.97, 95% CI 0.83–1.13, N_E_ = 7; Males: 1.15, 95% CI 0.72–1.83, N_E_ = 3) (Table 3, Fig. 2. For other types, see Additional file 1: Figs. S1, S2).

**Table 2:** Subgroup analyses of the association between baseline HPV-16 serostatus and HPV-16 incident detection

HPV-16	Subgroup	Females			Males		
		N^a^	Estimates [95% CI], I^2b^	*p* -value^c^	N^a^	Estimates [95% CI], I^2b^	*p-value*^c^
*(A) Primary outcomes*							
Incident detection		11	0.70 [0.61–0.80], 32%	NA	5	1.43 [1.12–1.83], 0%	NA
Persistent HPV positivity	6 months	4	0.58 [0.44–0.77], 1%	0.39	1	1.39 [0.80–2.41], NA	NA
	12 months	5	0.68 [0.56–0.83], 0%		NA		
Stratified by HIV status	HIV negative	1	1.68 [0.63–4.46], NA	0.31	1	1.70 [0.79–3.66], NA	0.64
	HIV positive	3	0.97 [0.64–1.48], 0%		1	1.00 [0.44–2.29], NA	
*(B) Subgroup analysis for incident detection*							
Detection site	Cervical	11	0.70 [0.44–1.12], 32%	NA	NA		0.81
	Penile/scrotal	NA			2	1.36 [1.01–1.83], 0%	
	Anal	NA			2	1.60 [0.99–2.56], 0%	
	Oral	NA			1	1.70 [0.50–5.90], NA	
Sexual orientation (among males)^d^	MSM	NA			5	1.30 [098–1.74], 0%	0.45
	MSW				2	1.41 [1.05–1.91], 0%	
	MSWM				1	0.88 [0.45–1.73], NA	
Age group^e^	≤ 30 years old	3	0.64 [0.49–0.84], 39%	0.61	1	1.15 [0.31–4.20], NA	0.55
	> 30 years old	6	0.84 [0.54–1.32], 55%		2	2.08 [0.99–4.40], 0%	
*(C) Sensitivity analysis for incident detection*							
Crude versus adjusted	Crude	8	0.69 [0.60–0.80], 47%	0.8	3	1.80 [0.94–3.43], 0%	0.45
	Adjusted	3	0.70 [0.58–0.80], 0%		2	1.38 [1.05–1.80], 0%	
Reported versus self-calculated	Reported	5	0.67 [0.58–0.78], 36%	0.53	5	1.43 [1.12–1.83], 0%	NA
	Self-calculated	6	0.70 [0.60–0.82], 40%		NA		
Measure type	IRR	3	0.64 [0.55–0.75], 35%	0.21	1	1.15 [0.3–4.20], NA	0.74
	HR	2	0.78 [0.58–1.06], 0%		4	1.44 [1.12–1.86], 0%	
	RR	4	0.88 [0.66–1.18], 0%		NA		
	OR	2	0.64 [0.44–0.92], 79%		NA		
Serologic assay^f^	Neutralizing	4	0.69 [0.38–1.25], 62%	0.92	NA		NA
	Non-neutralization	7	0.67 [0.59–0.76], 0%		5	1.43 [1.12–1.83], 0%	
DNA primer^g^	PGMY09/11	2	0.61 [0.47–0.79], 28%	<0.05	3	1.80 [0.94–3.43], 0%	0.76
	SPF10	5	0.70 [0.61–0.81], 0%		1	1.40 [0.81–2.42], NA	
	MY09/11	3	0.91 [0.67–1.22], 0%		NA		
Length of follow up	0–20 months	1	0.94 [0.35–2.54], NA	0.67	2	1.36 [0.82–2.25], 0%	0.93
	20–60 months	5	0.67 [0.58–0.78], 38%		1	1.70 [0.50–5.90], NA	
	60 + months	5	0.70 [0.59–0.82], 48%		2	1.48 [1.02–2.14], 12%	
Study quality	High	2	0.74 [0.54–1.01], 0%	0.76	1	2.34 [0.92–5.97], NA	0.29
	Medium	9	0.70 [0.59–0.82], 14%		4	2.38 [1.07–1.78], 19%	

^a^N = number of estimates pooled together. ^b^Between study heterogeneity was measured using the I2 statistic. ^c^Differences between subgroups were tested using the Q statistic (two-sided 5% alpha level). The *p* value is presented. ^d^MSM = Men who have sex with men; MSW = Men who have sex with women; MSWM = Men who have sex with men and women. ^e^Median/ mean age of the group of participants for each estimate was used. ^f^Neutralizing assay includes: Multiplex PsV-Luminex assay, cLIA, and SEAP-NA. Non-neutralizing assays includes: VLP-ELISA, Luminex-based multiplex serology assay. ^g^DNA primers sets have differing sensitivity of detection as follows: MY09/11 < SPF10 < PGMY09/11 (highest sensitivity to lowest). NA indicates when there is no estimate falling into the respective category (not able to pool)

**Table 3:** Subgroup analyses of the association between baseline HPV-18 serostatus and HPV-18 incident detection

HPV-18	Subgroup	Females			Males		
		N^a^	Estimates [95%CI], I^2b^	*p*-value^c^	N^a^	Estimates [95%CI], I^2b^	*p*-value^c^
(A)* Primary outcomes*							
Incident detection		7	0.97 [0.83–1.13], 0%	NA	3	1.15 [0.72–1.83], 0%	NA
Persistent detection	6 months	3	0.84 [0.45–1.58], 70%	0.99	1	0.19 [0.03–1.28], NA	NA
	12 months	4	0.84 [0.62–1.15], 0%		NA		
Stratified by HIV status	HIV negative	1	0.15 [0.02–1.16], NA	0.12	1	1.10 [0.40–3.06], NA	0.37
	HIV positive	2	0.41 [0.08–2.03], 66%		1	2.30 [0.73–7.22], NA	
(B)* Subgroup analysis for incident detection*							
Detection site	Cervical	7	0.97 [0.83–1.13], 0%	NA	NA		0.26
	Penile/scrotal	NA			2	0.93 [0.55–1.58], 0%	
	Anal	NA			1	1.50 [0.79–2.86], NA	
	Oral	NA			NA		
Sexual orientation (among men)^d^	MSM	NA			2	1.42 [0.78–2.61], 0%	0.71
	MSW				1	1.01 [0.51–2.01], NA	
	MSWM				1	1.02 [0.41–2.56], NA	
Age group ^e^	≤ 30 years old	2	0.95 [0.68–1.31], 0%	0.65	1	1.83 [0.18–18.39], NA	NA
	> 30 years old	4	0.61 [0.25–1.48], 71%		NA		
(C)* Sensitivity analysis for incident detection*							
Crude versus adjusted	Crude	5	1.00 [0.80–1.26], 0%	0.67	1	1.83 [0.18–28.39], NA	0.69
	Adjusted	2	0.94 [0.77–1.16], 0%		2	1.13 [0.69–1.85], 29%	
Reported versus self-calculated	Reported	3	0.93 [0.76–1.13], 0%	0.45	3	1.15 [0.72–1.83], 0%	NA
	Self-calculated	4	1.05 [0.82–1.33], 0%		NA		
Measure type	IRR	2	0.94 [0.78–1.14], 0%	0.67	1	1.83 [0.18–28.39], NA	0.67
	HR	1	0.95 [0.59–1.52], NA		2	1.13 [0.69–1.85], 29%	
	RR	3	116 [0.82–1.64], 0%		NA		
	OR	1	0.80 [0.44–1.46], NA		NA		
Serologic assay^f^	Neutralizing	1	1.02 [0.64–1.63], NA	0.83	NA		NA
	Non-neutralization	6	0.96 [0.82–1.13], 0%		3	1.15 [0.72–1.83], 0%	
DNA primer^g^	PGMY09/11	1	0.80 [0.44–1.46], NA	0.44	1	1.83 [0.18–28.39], NA	0.49
	SPF10	4	0.94 [0.79–1.12], 0%		1	1.50 [0.79–2.86], NA	
	MY09/11	2	1.19 [0.82–1.72], 2%		NA		
Length of follow up	0–20 months	NA		0.35	2	1.52 [0.2–2.83], 0%	0.21
	20–60 months	4	0.93 [0.78–1.11], 0%		NA		
	60+ months	3	1.09 [0.82–1.46], 0%		1	0.90 [0.53–1.55], NA	
Study quality	High	1	0.80 [0.44–1.46], NA	0.51	NA		NA
	Medium	6	0.98 [0.84–1.15], 0%		3	1.15 [0.72–1.83], 0%	

^a^N = number of estimates pooled together. ^b^Between study heterogeneity was measured using the I2 statistic. ^c^Differences between subgroups were tested using the Q statistic (two-sided 5% alpha level). The p-value is presented. ^d^MSM = Men who have sex with men; MSW = Men who have sex with women; MSWM = Men who have sex with men and women. ^e^Median/ mean age of the group of participants for each estimate was used. ^f^Neutralizing assay includes: Multiplex PsV-Luminex assay, cLIA, and SEAP-NA. Non-neutralizing assays includes: VLP-ELISA, Luminex-based multiplex serology assay. ^g^DNA primers sets have differing sensitivity of detection as follows: MY09/11 < SPF10 < PGMY09/11 (highest sensitivity to lowest). NA indicates when there is no estimate falling into the respective category (not able to pool)

Consistent with observed sex differences, we found a negative association for HPV-16 incident detection when restricting to cervical/cervicovaginal sites (0.70, 95% CI 0.44–1.12, N_E_ = 11). Among males, the associations remained positive when restricting to specific sites: penile/scrotal (1.36; 95% CI 1.01–1.83, N_E_ = 2), anal (1.60, 95% CI 0.99–2.56, N_E_ = 2), and oral sites (1.30, 95% CI 0.60–2.84, N_E_ = 2) (Table 2, Additional file 1: Fig. S4). However, differences by site among males were inconclusive for HPV-18 and with large confidence intervals (Table 3).

We did not find any significant differences in the direction and/or magnitude of association by sexual orientation for males, nor by age groups across study estimates of the associations between baseline HPV serostatus and type-specific incident detection for both HPV-16 and HPV-18 (Tables 2, 3, Additional file 1: Fig. S5).

### Association between HPV serostatus and type-specific persistent HPV positivity

Among females, we found some evidence of a protective effect of baseline antibody on HPV-16 persistent detection (6 months: 0.58, 95% CI 0.44–0.77, N_E_ = 4; 12 months: 0.68, 95% CI: 0.56–0.83, N_E_ = 5) (Tables 2, 3). Only one study estimate on persistent infection was available for males for HPV-16 (1.39, 95% CI 0.80–2.41) and HPV-18 (0.19, 95% CI 0.03–1.28) for the 6-month interval.

### Association between HPV antibody level and incident HPV detection

**Fig. 3:**
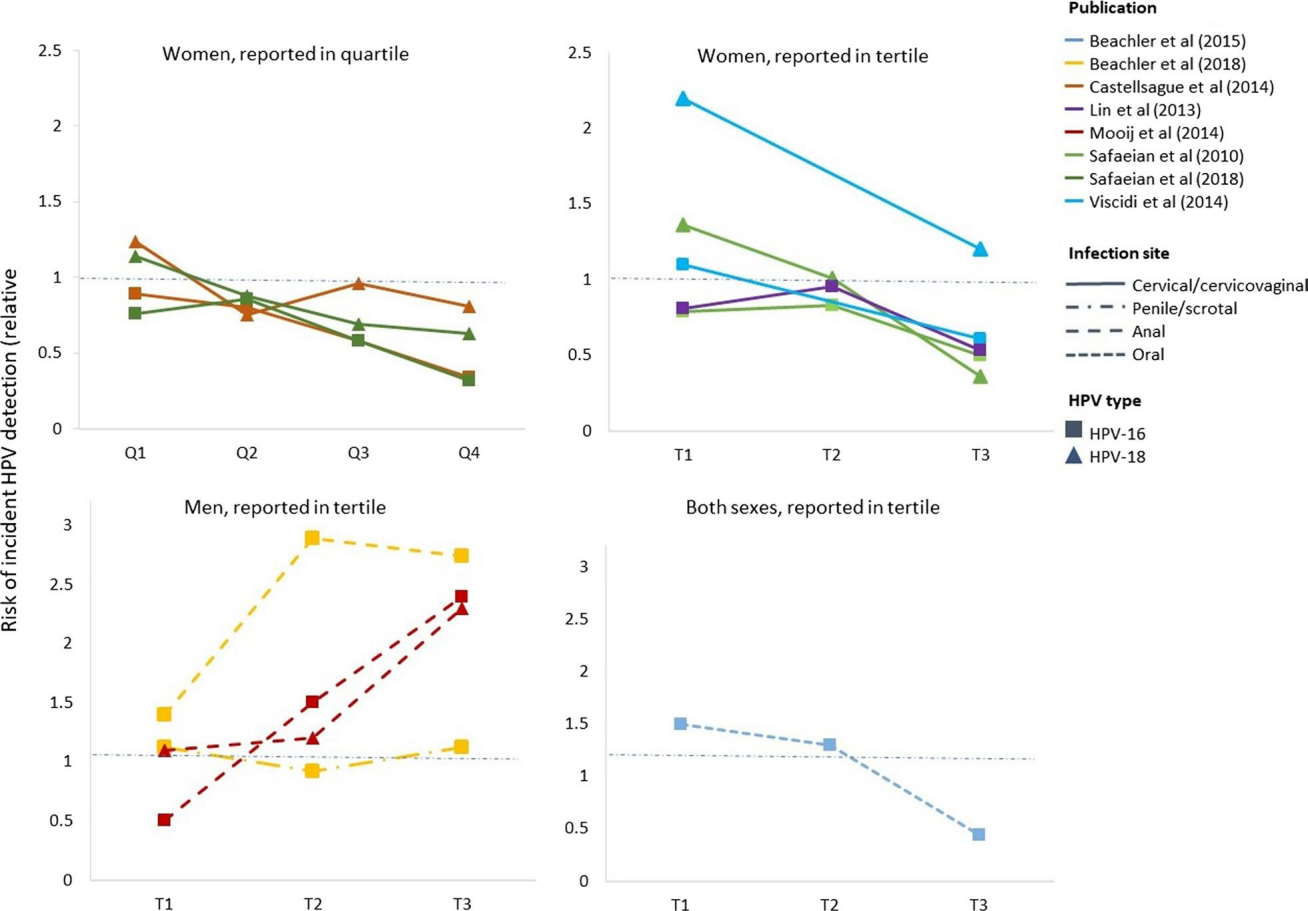
Trends in estimates of association between baseline HPV serostatus and type-specific HPV incident detection by HPV antibody concentration level, stratified by sex (females, males, or both). Seven publications measured antibody levels in terms of ELISA units per mL (EU/mL) [14, 19, 21, 22, 24, 25, 41], and one, in Luminex units per mL (LU/mL) [15]. Five studies reported estimates by tertiles of antibody levels [14, 15, 22, 24, 41], two by quartiles [21, 24], and one dichotomized antibody levels (low versus high concentration) [19]. T1, T2, T3 refer to the lowest, middle, and upper tertiles of antibody levels used by each publication, respectively; Q1, Q2, Q3, Q4 refer to the first, second, third, and fourth quartiles, respectively; Low and High refer to low and high antibody concentrations, respectively. Horizontal dashed lines indicate the null value of the estimate of the association (1 on a relative scale). The y-axis indicates the risk of incident HPV detection on the relative scale. For instance, a blue dot with the highest y-value in the top right panel indicates a risk ratio of incident detection between individuals with no HPV antibodies versus individuals with naturally acquired HPV antibodies in the lowest tertile. The lower end of antibody level for the lowest tercile/quartile used by publications was between 7–8 EU/mL or 0.2–0.28 LU/mL, and the higher end of between 40–64 EU/mL or 0.46–0.55 LU/mL

A total of eight publications reported estimates of association by antibody levels for HPV-16 and HPV-18 separately for males and females. Compared to the lowest antibody titres categories, the highest ones were associated with reduced likelihood of HPV detection among females for HPV-16 (0.65, 95% CI 0.55–0.76, N_E_ = 5) and for HPV-18 (0.55, 95% CI 0.41–0.75, N_E_ = 4). Out of nine reported estimates of association among female participants for incident detection at cervical/cervicovaginal site, 4 estimates for HPV-16 and 4 estimates for HPV-18 showed negative associations between increasing baseline antibody levels and the likelihood of HPV detection. We found signs of a positive association between the highest antibody threshold categories and HPV detection risk among males for HPV-16 (1.93, 95% CI 0.67–5.57, N_E_ = 2), however, the results were inconclusive. There was only one study reporting males’ estimates by tertiles for HPV-18 (Fig. 3, Additional file 1: Fig. S6). Finally, one estimate of association among both male and female participants combined, measured at oral sites for HPV-16 suggested a negative trend (Fig. 3).

### Impact of HIV status on HPV serostatus and type-specific incident HPV detection

**Fig. 4:**
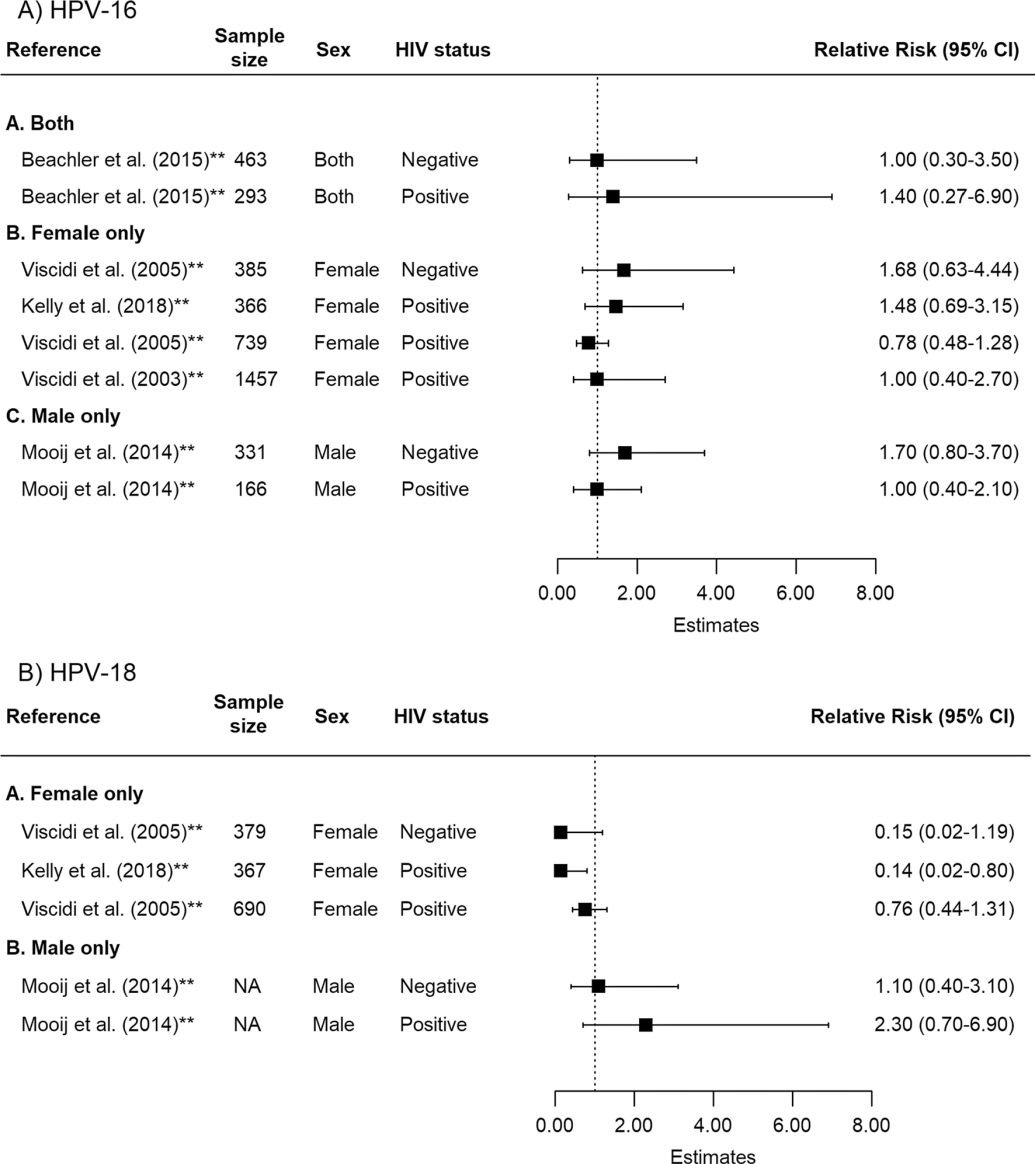
Forest plot of the association between baseline HPV serostatus and type-specific HPV incident detection by HIV status for **A** HPV-16 and **B** HPV-18. †Indicates estimates that are adjusted for confounders; NA = Not available. Participants included in the publication by Kelly et al. [40] were restricted to women living with HIV. The other publications reported separate estimates of measures of association by HIV status. CD4 cell counts of HIV positive individuals were reported in varying format by publications (threshold, median). All 5 publications adjusted for the baseline CD4 cell counts in the calculation of estimates in either main analysis or sensitivity analysis. Only two publications reported information regarding treatment status of HIV positive individuals at baseline and/or throughout the study period

Results from publications assessing the associations between HPV serostatus and incident type-specific HPV detection by baseline HIV status were inconclusive. For HPV-16, the estimate of association between baseline serostatus and subsequent incident HPV detection was 1.68 (95% CI 0.63–4.46, N_E_ = 1) among female individuals without HIV and 0.97 (95% CI 0.64–1.48, N_E_ = 3) among female individuals living with HIV. For HPV-18, the results were also not statistically significant (female individuals living without HIV: 0.15, 95% CI 0.02–1.16, N_E_ = 1; women living with HIV: 0.41, 95% CI 0.08–2.03, N_E_ = 2) (Fig. 4, Tables 2, 3). Only one study reported estimates for male individuals living without HIV (HPV-16: 1.70, 95% CI 0.79–3.66; HPV-18: 1.10, 95% CI 0.40–3.06) and for male individuals living with HIV (HPV-16: 1.00, 95% CI 0.44–2.29; HPV-18: 2.30, 95% CI 0.73–7.22) (Table 2). Included studies did not provide enough information about the HIV disease stage or treatment status to perform subgroup analyses.

### Sensitivity analyses

For both HPV-16 and HPV-18 incident detections, we did not observe large differences between crude and adjusted estimates, methods used for serotyping, and by study quality, after stratifying by sex (Tables 2, 3, Additional file 1: Figs. S7, S8). The only difference was found in the primer sets used for HPV DNA detection among females for HPV-16 (PGMY09/11: 0.61, 95% CI 0.47–0.79, N_E_ = 2; SPF10: 0.70, 95% CI 0.61–0.81, N_E_ = 5; MY09/11: 0.91, 95% CI 0.67–1.22, N_E_ = 3).

Leave-one-out analyses did not identify any individual study estimate that strongly impacted the pooled estimates of the association or the measured heterogeneity for both HPV-16 and HPV-18 (Additional file 1: Fig. S9). Finally, funnel plots for all HPV types as well as Egger’s test for HPV-16 and HPV-18 showed no evidence of publication bias (HPV-16 Egger P = 0.17; HPV-18 Egger P = 0.78; Additional file 1: Fig. S10).

## Discussion

Pooling data on 62,363 participants, this systematic review found evidence of protective effects of baseline HPV seropositivity and subsequent detection of incident and persistent HPV, mainly for HPV-16 among females, but not among males, nor for HPV-18 or other HPV types. Moreover, our results suggested a protective effect of higher antibody levels against subsequent HPV-16 detection at cervical/cervicovaginal sites among female subjects, but not among males. Based on the 5 published studies available, there was no evidence of a differential impacts of naturally acquired antibodies on subsequent HPV-16/18 detection by HIV status.

Even with increasing uptake and coverage of HPV vaccines, improving our understanding of the heterogenous effects of naturally acquired antibodies on reinfection and persistent infection is important, especially to improve structural assumptions and parameterization of mathematical models of HPV, for instance. Our results suggest a partially protective effect of natural antibodies among females, but this level of protection is much lower than the one conferred by vaccines. For example, trial data show that a single dose of HPV 16/18 vaccine results in antibody geometric mean titres 5–9 times larger than the protection acquired from natural infection, even several years after vaccination [42].

Consistent with previous studies, the current meta-analysis found important sex differences [29]. The finding that, among males, HPV seropositivity increases risks of subsequent HPV detection needs to be interpreted cautiously. First, it is possible that the estimates are confounded by partially or unmeasured variables, such as sexual behaviours, as some of our included estimates were unadjusted in the original studies. Further, differences in immune response in extracervical sites, and its potential in explaining the sex differentials, requires further research. Several studies have noted differences in the level of immune response introduced after an infection at a mucosal epithelium of female genitals compared to the immune response after an infection at keratinized epithelium of male genitals (i.e., penis), suggesting a higher viral antigen level and a stronger antibody response at mucosa [60, 61]. MSM could be more frequently exposed to HPV through anal mucosa than men who have sex with women. However, our site-specific analyses do not show a reduced risk of subsequent HPV detection at anal sites in stratified analyses among MSM.

Besides a thorough examination of incident detections, we examined the impact of naturally acquired antibody on persistent HPV positivity. Similar to the association between baseline HPV serostatus and a subsequent incident detection (one time detection of HPV-DNA), there was a reduction in risk of persistent HPV positivity (two or more detections of HPV-DNA during consecutive follow-ups). Current knowledge points to multiple interactions between HPV infection and HIV status [30, 31, 40, 43, 44]. Our results remained inconclusive with wide confidence intervals in terms of the association between HPV antibodies and subsequent HPV detection by HIV status. Results stratified by HIV status were subject to limited precision due to a small number of estimates available for pooling as well as sources of unmeasured heterogeneity, such as antiretroviral treatment status and CD4 cell counts among PLHIV, that remained unadjusted due to low number of studies that measured these variables, which might have contributed to residual confounding.

Assessing the association between naturally acquired HPV antibodies and subsequent re-infection, as well as synthesizing the evidence of this association, are subject to challenges and limitations. First, it is challenging to differentiate between true re-infection and detection of an activated latent HPV or deposition when HPV DNA is detected [45–47]. Therefore, our results from pooled analysis require cautious interpretations when inferring about the role of natural history of HPV. Moreover, there is always a risk of misclassification of exposure and/or outcomes, depending on the type of serologic assay (i.e., neutralizing versus non-neutralizing) and DNA primer sets used for individual studies. Variability in types of serological tests or DNA primer sets used and difference in sensitivity by HPV type may have influenced the risk of misclassification in individual study results and introduced some degree of heterogeneity in the magnitude of the pooled estimates (rather than the direction of the association). A careful interpretation of the effect sizes while contextualizing the heterogeneity in study designs, population, and sensitivity of methodology used is required. Second, our analysis was restricted to type-specific risk of an incident detection and did not assess the effect of cross-protection of antibodies across different HPV types. Studies have found that infections with multiple HPV types was associated with persistence of HPV infections, while others did not find any differences [48, 49]. Third, some of our analyses had limited statistical power due to a small number of publications reporting estimates among men, MSM, by HIV serostatus, and age. Lastly, our study could not rule out the potential information bias and selection bias, as some publications did not always report characteristics of study populations such as mean/median age in a standardized format, nor participation rate/loss to follow up. Our estimates are also subject to confounding, as we pooled adjusted and unadjusted estimates provided by individual studies to increase the number of effect sizes pooled.

This review synthesises current knowledge on naturally acquired immunity against HPV. It substantially increases the precision of effect sizes estimates, especially for HPV-16. Compared to the previous review, we added 12 additional publications and nearly 35,000 new participants to the pooled analysis. Our results suggest differences in the effect of HPV antibodies between males and females and a potential negative dose–response relationship between antibody titre levels and subsequent detection for HPV-16 and HPV-18 in females. Although our results were inconclusive regarding differential impacts of naturally acquired immunity by HIV status, PLHIV could be at greater risk of reinfection, and consequently, at higher risk of developing HPV-attributable cancers.

## Conclusion

We did not find evidence that naturally acquired HPV antibodies protect against subsequent HPV positivity in males and provide only modest protection among females for HPV-16. Being the first study to evaluate the dose–response relationship between antibody titre levels and subsequent detection of HPV-DNA, we found some evidence of a potential negative dose–response. Finally, we did not find conclusive evidence of differential impacts of naturally acquired antibodies by HIV status, and further investigation is warranted.

### Supplementary Information

**Additional file 1.** Association of naturally acquired type-specific HPV antibodies and subsequent HPV re-detection: Systematic review and meta-analysis.

## Data Availability

All relevant data are included in the article or uploaded as additional information.
