# Supplementary material for: Association of naturally acquired type-specific HPV antibodies and subsequent HPV re-detection: systematic review and meta-analysis
Source: Infect Agent Cancer. 2023 Nov 8;18:70. doi: 10.1186/s13027-023-00546-3 (PMC10631102; doi:10.1186/s13027-023-00546-3)
Supplement: Supplementary file 1 — Additional file 1. Association of naturally acquired type-specific HPV antibodies and subsequent HPV re-detection: Systematic review and meta-analysis. [file 13027_2023_546_MOESM1_ESM.docx]

**Supplementary materials**

**Supplementary Table 1. Prisma checklist**

| **Section and Topic** | **Item #** | **Checklist item** | **Location where item is reported** |
| --- | --- | --- | --- |
| **TITLE** | | |  |
| Title | 1 | Identify the report as a systematic review. | 2 |
| **ABSTRACT** | | |  |
| Abstract | 2 | See the PRISMA 2020 for Abstracts checklist. | 2 |
| **INTRODUCTION** | | |  |
| Rationale | 3 | Describe the rationale for the review in the context of existing knowledge. | 3 |
| Objectives | 4 | Provide an explicit statement of the objective(s) or question(s) the review addresses. | 4 |
| **METHODS** | | |  |
| Eligibility criteria | 5 | Specify the inclusion and exclusion criteria for the review and how studies were grouped for the syntheses. | 4 |
| Information sources | 6 | Specify all databases, registers, websites, organisations, reference lists and other sources searched or consulted to identify studies. Specify the date when each source was last searched or consulted. | 4 |
| Search strategy | 7 | Present the full search strategies for all databases, registers and websites, including any filters and limits used. | 4 |
| Selection process | 8 | Specify the methods used to decide whether a study met the inclusion criteria of the review, including how many reviewers screened each record and each report retrieved, whether they worked independently, and if applicable, details of automation tools used in the process. | 4 |
| Data collection process | 9 | Specify the methods used to collect data from reports, including how many reviewers collected data from each report, whether they worked independently, any processes for obtaining or confirming data from study investigators, and if applicable, details of automation tools used in the process. | 4 |
| Data items | 10a | List and define all outcomes for which data were sought. Specify whether all results that were compatible with each outcome domain in each study were sought (e.g. for all measures, time points, analyses), and if not, the methods used to decide which results to collect. | 4 |
|  | 10b | List and define all other variables for which data were sought (e.g. participant and intervention characteristics, funding sources). Describe any assumptions made about any missing or unclear information. | 4 |
| Study risk of bias assessment | 11 | Specify the methods used to assess risk of bias in the included studies, including details of the tool(s) used, how many reviewers assessed each study and whether they worked independently, and if applicable, details of automation tools used in the process. | 5-6 |
| Effect measures | 12 | Specify for each outcome the effect measure(s) (e.g. risk ratio, mean difference) used in the synthesis or presentation of results. | 5-6 |
| -Synthesis methods | 13a | Describe the processes used to decide which studies were eligible for each synthesis (e.g. tabulating the study intervention characteristics and comparing against the planned groups for each synthesis (item #5)). | 5-6 |
|  | 13b | Describe any methods required to prepare the data for presentation or synthesis, such as handling of missing summary statistics, or data conversions. | 5-6 |
|  | 13c | Describe any methods used to tabulate or visually display results of individual studies and syntheses. | 5-6 |
|  | 13d | Describe any methods used to synthesize results and provide a rationale for the choice(s). If meta-analysis was performed, describe the model(s), method(s) to identify the presence and extent of statistical heterogeneity, and software package(s) used. | 5-6 |
|  | 13e | Describe any methods used to explore possible causes of heterogeneity among study results (e.g. subgroup analysis, meta-regression). | 5-6 |
|  | 13f | Describe any sensitivity analyses conducted to assess robustness of the synthesized results. | 5-6 |
| Reporting bias assessment | 14 | Describe any methods used to assess risk of bias due to missing results in a synthesis (arising from reporting biases). | 5-6 |
| Certainty assessment | 15 | Describe any methods used to assess certainty (or confidence) in the body of evidence for an outcome. | 5-6 |
| **RESULTS** | | |  |
| Study selection | 16a | Describe the results of the search and selection process, from the number of records identified in the search to the number of studies included in the review, ideally using a flow diagram. | 6-7 |
|  | 16b | Cite studies that might appear to meet the inclusion criteria, but which were excluded, and explain why they were excluded. | 6-7 |
| Study characteristics | 17 | Cite each included study and present its characteristics. | 6-7 |
| Risk of bias in studies | 18 | Present assessments of risk of bias for each included study. | 6-7 |
| Results of individual studies | 19 | For all outcomes, present, for each study: (a) summary statistics for each group (where appropriate) and (b) an effect estimate and its precision (e.g. confidence/credible interval), ideally using structured tables or plots. | 7-8 |
| Results of syntheses | 20a | For each synthesis, briefly summarise the characteristics and risk of bias among contributing studies. | 7-8 |
|  | 20b | Present results of all statistical syntheses conducted. If meta-analysis was done, present for each the summary estimate and its precision (e.g. confidence/credible interval) and measures of statistical heterogeneity. If comparing groups, describe the direction of the effect. | 7-8 |
|  | 20c | Present results of all investigations of possible causes of heterogeneity among study results. | 7-8 |
|  | 20d | Present results of all sensitivity analyses conducted to assess the robustness of the synthesized results. | 7-8 |
| Reporting biases | 21 | Present assessments of risk of bias due to missing results (arising from reporting biases) for each synthesis assessed. | 7-8 |
| Certainty of evidence | 22 | Present assessments of certainty (or confidence) in the body of evidence for each outcome assessed. | 7-8 |
| **DISCUSSION** | | |  |
| Discussion | 23a | Provide a general interpretation of the results in the context of other evidence. | 8-10 |
|  | 23b | Discuss any limitations of the evidence included in the review. | 8-10 |
|  | 23c | Discuss any limitations of the review processes used. | 8-10 |
|  | 23d | Discuss implications of the results for practice, policy, and future research. | 8-10 |
| **OTHER INFORMATION** | | |  |
| Registration and protocol | 24a | Provide registration information for the review, including register name and registration number, or state that the review was not registered. | NA |
|  | 24b | Indicate where the review protocol can be accessed, or state that a protocol was not prepared. | NA |
|  | 24c | Describe and explain any amendments to information provided at registration or in the protocol. | NA |
| Support | 25 | Describe sources of financial or non-financial support for the review, and the role of the funders or sponsors in the review. | 11 |
| Competing interests | 26 | Declare any competing interests of review authors. | 11 |
| Availability of data, code and other materials | 27 | Report which of the following are publicly available and where they can be found: template data collection forms; data extracted from included studies; data used for all analyses; analytic code; any other materials used in the review. | 12- |

**Supplementary Table 2. Search strategy for the systematic review and meta-analysis** of the association between baseline HPV serostatus and type-specific HPV redetection**.**

| Database | Key words | No. of hits (Language) |
| --- | --- | --- |
| Medline (Pubmed) | ("Papillomaviridae"[Mesh] OR "HPV"[Title/Abstract] OR "papillomavi*"[Title/Abstract])  **AND**  ("Epidemiologic Studies"[Mesh] OR "Epidemiologic Stud*"[Title/Abstract] OR "Observational Studies as Topic"[Mesh] OR "Observational Stud*"[Title/Abstract] OR "Seroepidemiologic Studies"[Mesh] OR "Seroepidemiologic Stud*"[Title/Abstract] OR "Cohort Studies"[Mesh] OR "Cohort"[Title/Abstract] OR "Longitudinal Studies"[Mesh] OR "Longitudinal Stud*"[Title/Abstract] OR "Follow-Up Studies"[Mesh] OR "Follow-Up"[Title/Abstract] OR "Prospective Studies"[Mesh] OR "Prospective*"[Title/Abstract] OR "Clinical Study" [Publication Type] OR "Clinical Stud*" [Title/Abstract] OR "Clinical Trials as Topic"[Mesh] OR "Clinical Trial*"[Title/Abstract] OR "Randomized Controlled Trial"[Publication Type] OR "Randomized Controlled Trials as Topic"[Mesh] OR "Randomized Controlled Trial"[Title/Abstract])  **AND**  ("Antibodies, Viral"[Mesh] OR "Viral Antibodies"[Title/Abstract] OR "Antibodies, Neutralizing"[Mesh] OR "Neutralizing Antibod*"[Title/Abstract] OR "Papillomavirus Vaccines"[Mesh] OR "Papillomavirus Vaccin*"[Title/Abstract] OR "Serologic tests"[Mesh] OR "seropos*"[Title/Abstract] OR "seroreact*"[Title/Abstract] OR "serolog*"[Title/Abstract])  **AND**  ("DNA, Viral"[Mesh] OR "Viral DNA"[Title/Abstract] OR "Papillomavirus Infections"[Mesh] OR "Papillomavirus Infect*"[Title/Abstract] OR "infect*"[Title/Abstract] OR "reinfect*"[Title/Abstract] OR "re-infect*"[Title/Abstract])  **NOT**  ("Cross-Sectional Studies"[Mesh] OR "cross-sectional"[Title/Abstract] OR "cost-effectiveness") | 2089 (EN, FR, JP) |
| Embase | exp Papillomaviridae/ OR papillomavi*.ab,kw,ti OR HPV.ab,kw,ti  **AND**  exp epidemiology/ OR epidemiologic stud*.ab,kw,ti OR exp observational study/ OR observational stud*.ab,kw,ti OR exp seroepidemiology/ OR seroepidemiologic stud*.ab,kw,ti OR exp cohort analysis/ OR cohort.ab,kw,ti OR longitudinal stud*.ab,kw,ti OR exp follow up/ or follow-up.ab,kw,ti OR prospective*.ab,kw,ti OR exp clinical study/ OR clinical stud*.ab,kw,ti OR clinical trial*.ab,kw,ti OR randomized controlled trial.ab,kw,ti  **AND**  exp virus antibody/ OR viral antibodies.ab,kw,ti OR exp neutralizing antibody/ OR neutralizing antibod*.ab,kw,ti OR exp wart virus vaccine/ OR papillomavirus vaccin*.ab,kw,ti OR exp serology/ OR serologic stud*.ab,kw,ti OR seropos*.ab,kw,ti OR seroreact*.ab,kw,ti OR serolog*.ab,kw,ti  **AND**  exp virus DNA/ OR viral DNA.ab,kw,ti OR exp papillomavirus infection/ OR papillomavirus infect*.ab,kw,ti OR reinfect*.ab,kw,ti or re-infect.ab,kw,ti  **NOT**  exp cross-sectional study/ OR cross-sectional.ab,kw,ti OR cost-effectiveness.ab,kw,ti | 3711 (EN**)** |

**Supplementary Table 3**. Study quality assessment based on the modified Newcastle-Ottawa Scale

|  | Selection | | | | Comparability | Outcome | | |  |  |  |
| --- | --- | --- | --- | --- | --- | --- | --- | --- | --- | --- | --- |
| **Author (Year)** | **Sampling** | **Non-respondents** | **Assessment of exposure** | **Outcome not present at baseline** | **Comparability of cohorts** | **Assessment of outcome** | **Adequate follow-up period** | **Lost to FU** | **Total stars** | **Quality** |  |
|  |  |  |  |  |  |  |  |  |  |  |  |
| Beachler et al (2015) | c | d | a (**) | a (*) | a (*) | a (***) | a (*) | d | 8 | Medium |  |
| Beachler et al (2018) | c | b (*) | a (**) | a (*) | a (*) | a (***) | a (*) | b (*) | 10 | High |  |
| Castellsague et al (2014) | d | d | a (**) | a (*) | a (*) | b (**) | a (*) | b (*) | 8 | Medium |  |
| Eldridge et al (2017) | c | d | a (**) | a (*) | a (*) | a (***) | a (*) | c | 8 | Medium |  |
| Herrero et al (2011) | a (*) | d | a (**) | a (*) | b | b (**) | a (*) | d | 7 | Medium |  |
| Kelly et al (2018) | c | d | b (*) | a (*) | a (*) | b (**) | a (*) | b (*) | 7 | Medium |  |
| Konno et al (2014) | d | d | a (**) | a (*) | b | b (**) | a (*) | d | 6 | Medium |  |
| Lin et al (2013) | b (*) | d | a (**) | a (*) | b | b (**) | a (*) | d | 7 | Medium |  |
| Lu et al (2010) | c | b (*) | a (**) | a (*) | b | a (***) | a (*) | c | 8 | Medium |  |
| Lu et al (2012) | c | b (*) | a (**) | a (*) | a (*) | a (***) | a (*) | c | 9 | High |  |
| Mooij et al (2014) | c | d | a (**) | a (*) | a (*) | b (**) | a (*) | d | 7 | Medium |  |
| Moscicki et al (2013) | c | d | a (**) | a (*) | b | a (***) | a (*) | d | 7 | Medium |  |
| Pamnani et al (2016) | c | d | a (**) | a (*) | a (*) | d | a (*) | d | 5 | Medium |  |
| Pierce Campbell et al (2016) | c | d | a (**) | a (*) | b | a (***) | a (*) | d | 7 | Medium |  |
| Robbins et al (2014) | b (*) | d | a (**) | a (*) | a (*) | b (**) | a (*) | d | 8 | Medium |  |
| Rosillon et al (2019) | a (*) | d | a (**) | a (*) | b | b (**) | a (*) | d | 7 | Medium |  |
| Safaeian et al (2010) | a (*) | d | a (**) | a (*) | a (*) | b (**) | a (*) | d | 8 | Medium |  |
| Safaeian et al (2018) | a (*) | d | a (**) | a (*) | a (*) | b (**) | a (*) | d | 8 | Medium |  |
| Szarewski et al (2012) | a (*) | d | a (**) | a (*) | b | b (**) | a (*) | d | 7 | Medium |  |
| Triglav et al (2017) | b (*) | d | b (*) | a (*) | a (*) | d | a (*) | c | 5 | Medium |  |
| Viscidi et al (2003) | b (*) | d | a (**) | a (*) | b | c (*) | a (*) | c | 6 | Medium |  |
| Viscidi et al (2004) | b (*) | b (*) | a (**) | a (*) | b | c (*) | a (*) | c | 7 | Medium |  |
| Viscidi et al (2005) | c | d | a (**) | a (*) | a (*) | c (*) | a (*) | c | 6 | Medium |  |
| Wentzensen et al (2011) | b (*) | d | a (**) | a (*) | a (*) | c (*) | a (*) | d | 7 | Medium |  |
| Wilson et al (2014) | d | d | a (**) | a (*) | a (*) | a (***) | a (*) | b (*) | 9 | High |  |
| Yao et al. (2021) | b (*) | d | a (**) | a (*) | a (*) | b (**) | a (*) | b (*) | 9 | High |  |

* = stars given to each category. a, b, c, or d were assigned based on the criteria outlined in Supplementary information 3.

**
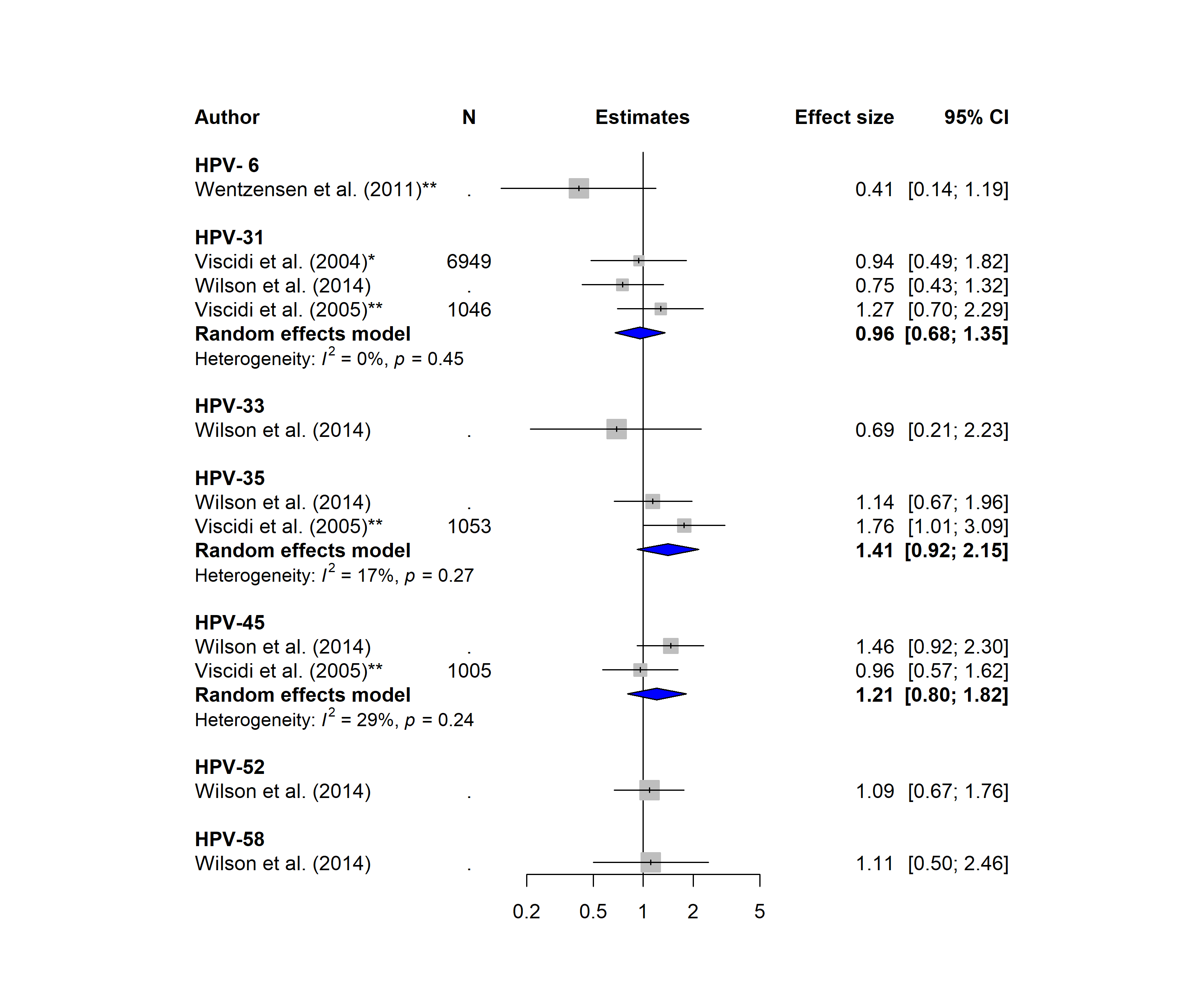
**

**Supplementary Figure 1.** Forest plot of the association between baseline HPV serostatus and type-specific HPV incident infection by HPV type among females(other types).

*indicates estimates that are self-calculated using the data presented in each publication; ** indicates estimates that are adjusted for confounders; NA=not available.


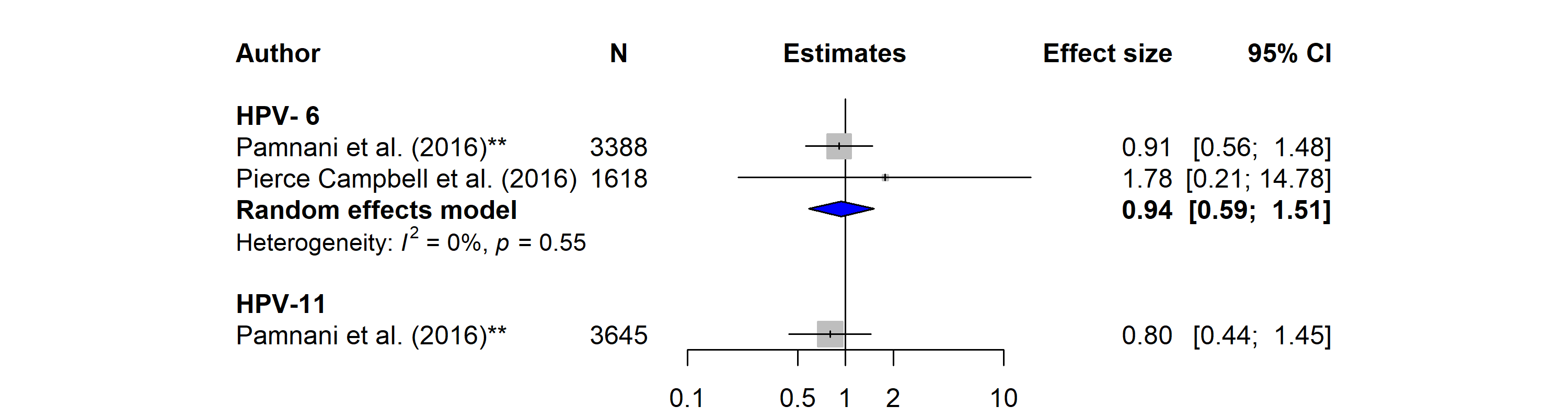


**Supplementary Figure 2.** Forest plot of the association between baseline HPV serostatus and type-specific HPV incident infection by HPV type among males (other types)

*indicates estimates that are self-calculated using the data presented in each publication; ** indicates estimates that are adjusted for confounders; NA=not available.

**
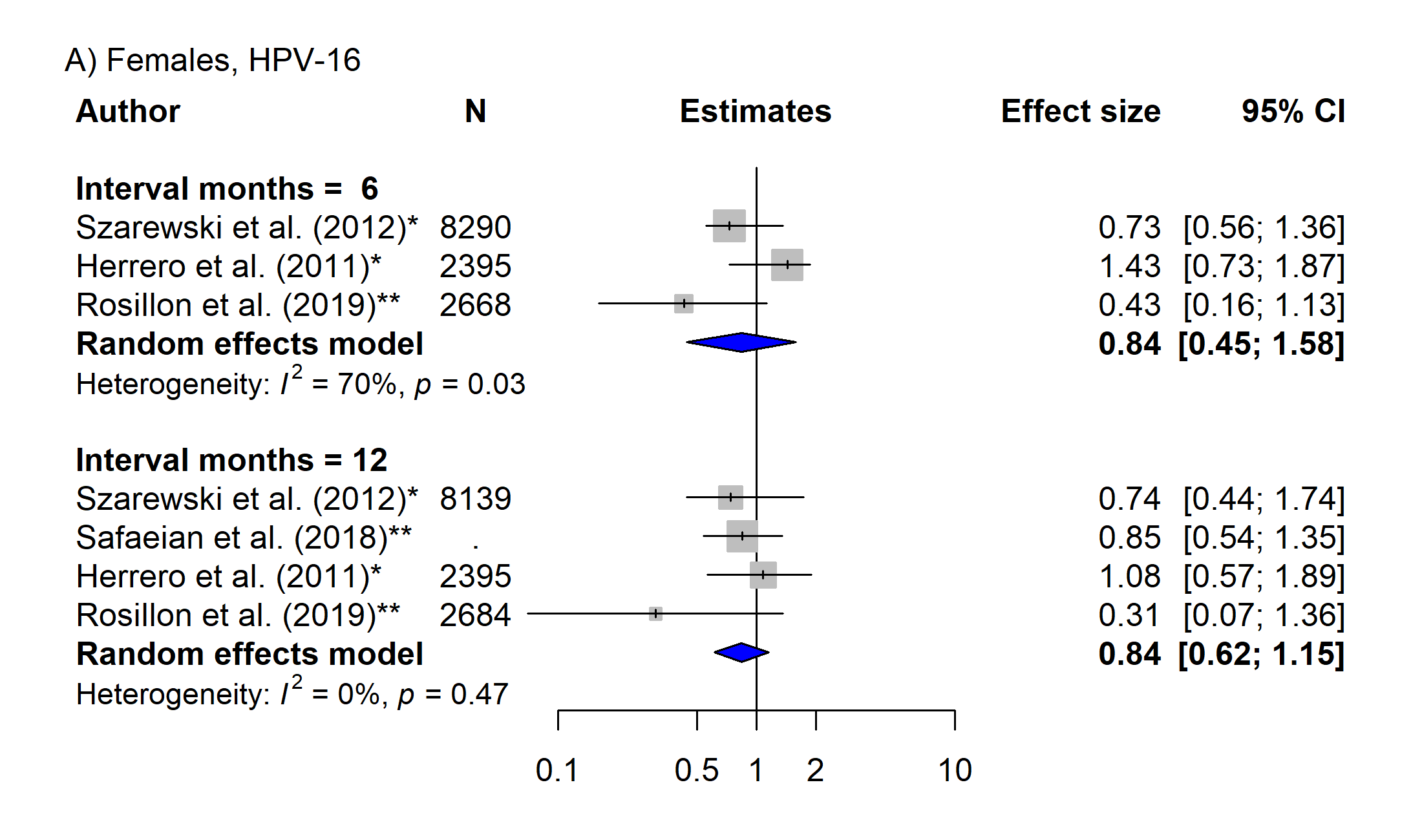
**

**
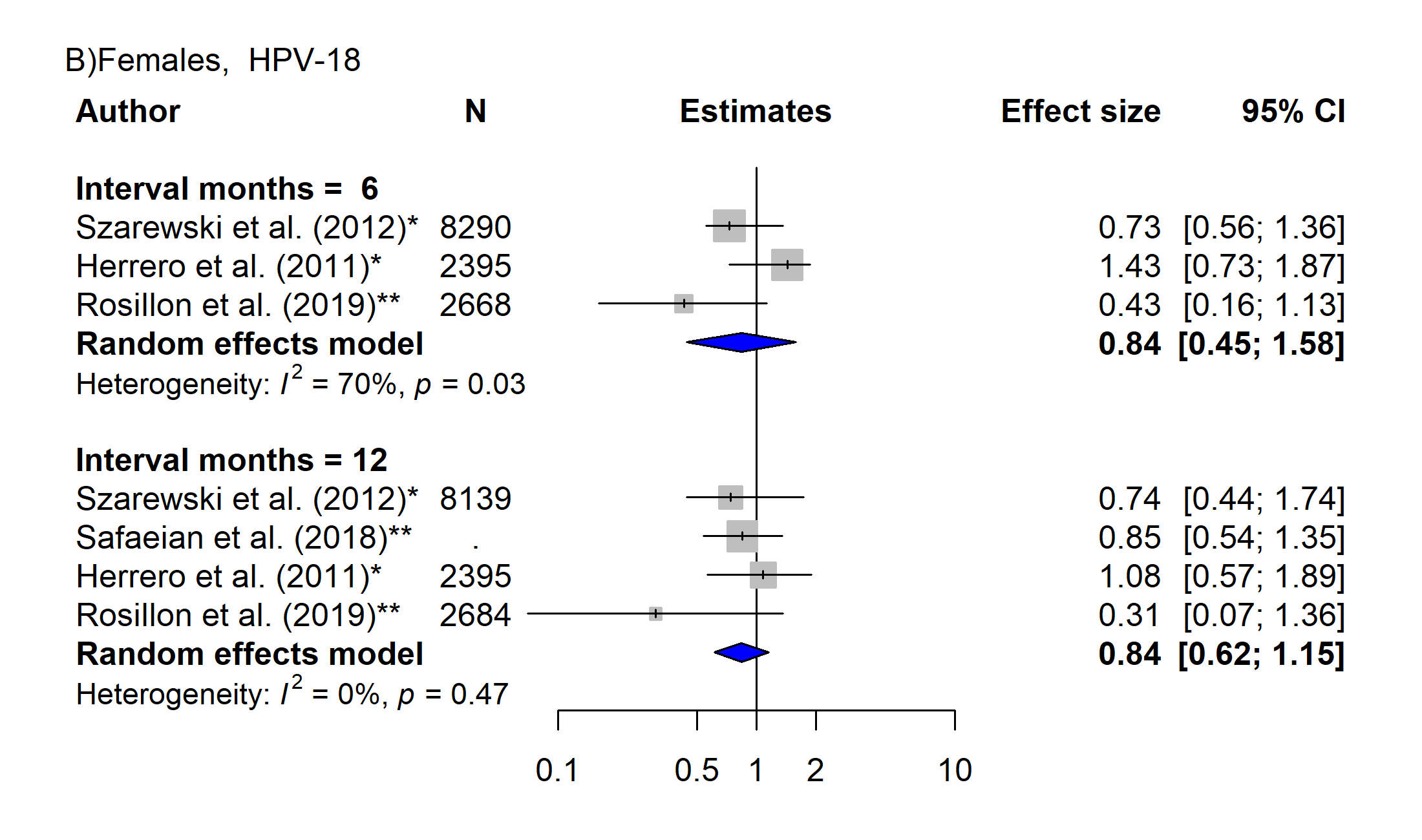
**

**Supplementary Figure 3.** Forest plot of the association between baseline HPV serostatus and type-specific HPV persistent HPV positivity by time interval of HPV DNA assessment for females, A) HPV-16 and B) HPV-18.

* indicates estimates that are self-calculated using the data presented in each publication; ** indicates estimates that are adjusted for confounders; NA=not available.

**
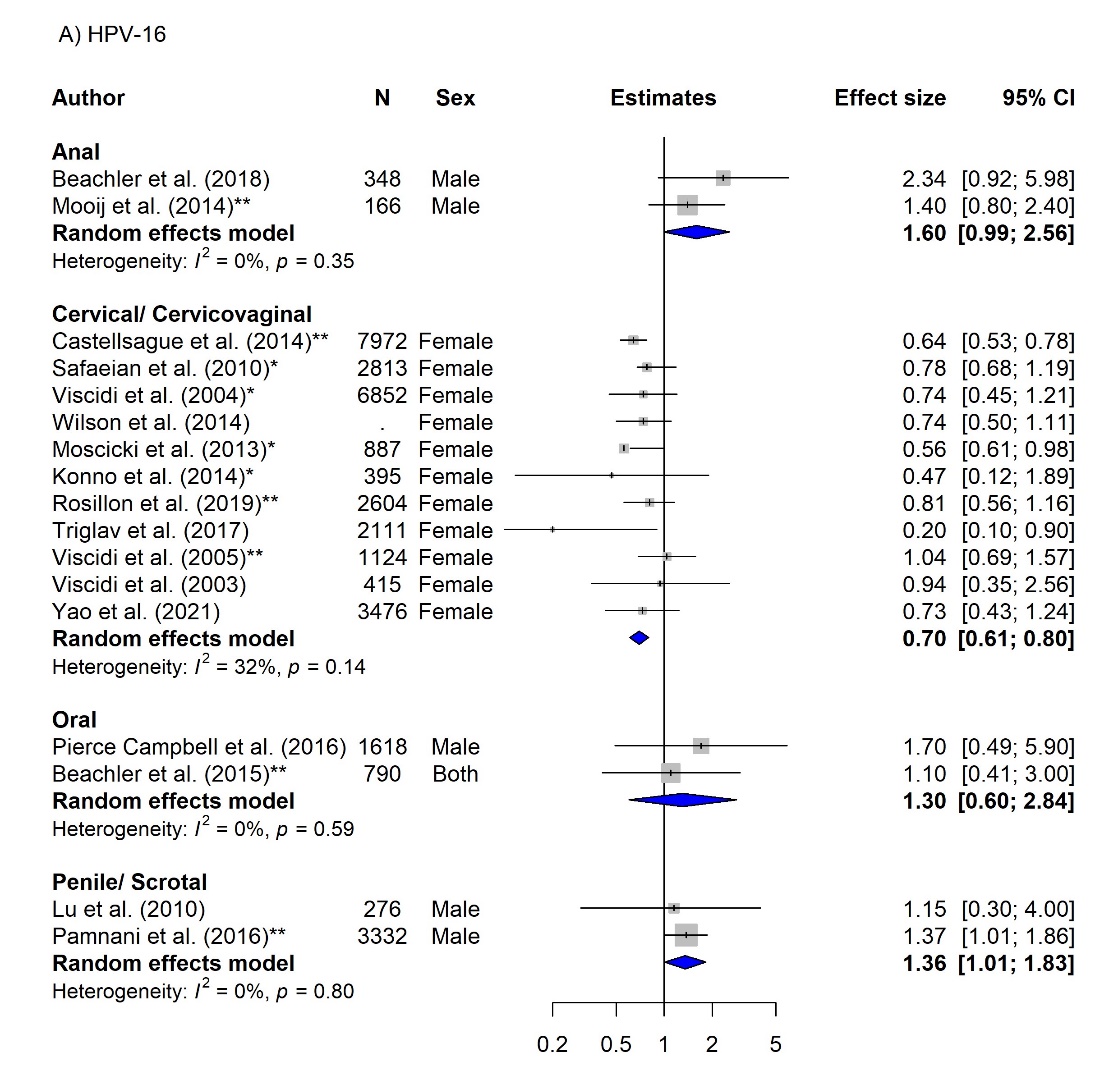

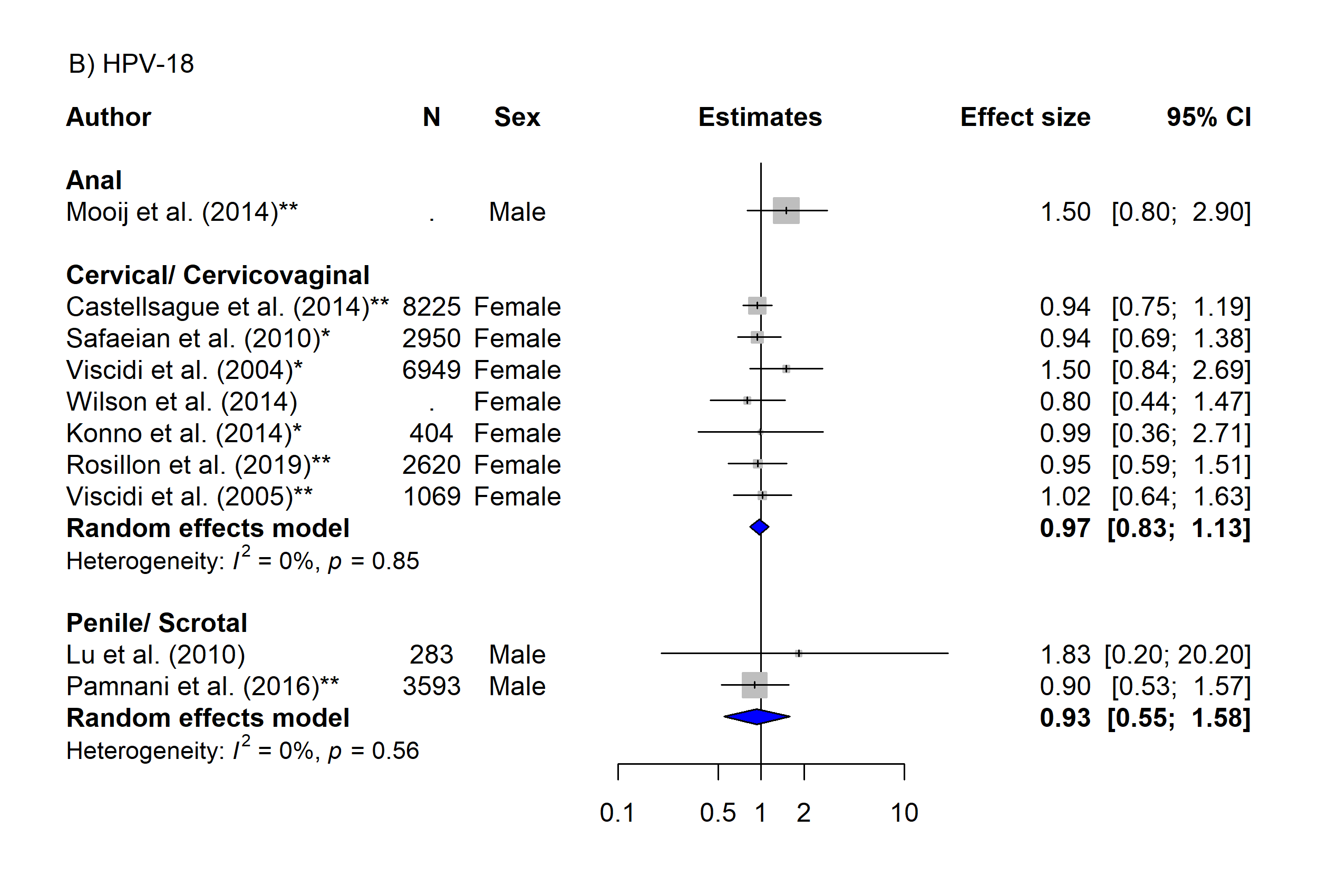
**

**Supplementary Figure 4**. Forest plot of the association between baseline HPV serostatus and type-specific HPV incident infection by infection site for A) HPV-16 and B) HPV-18.

*indicates estimates that are self-calculated using the data presented in each publication; ** indicates estimates that are adjusted for confounders; NA=not available. Estimates from oral site were measured among male subjects.

**
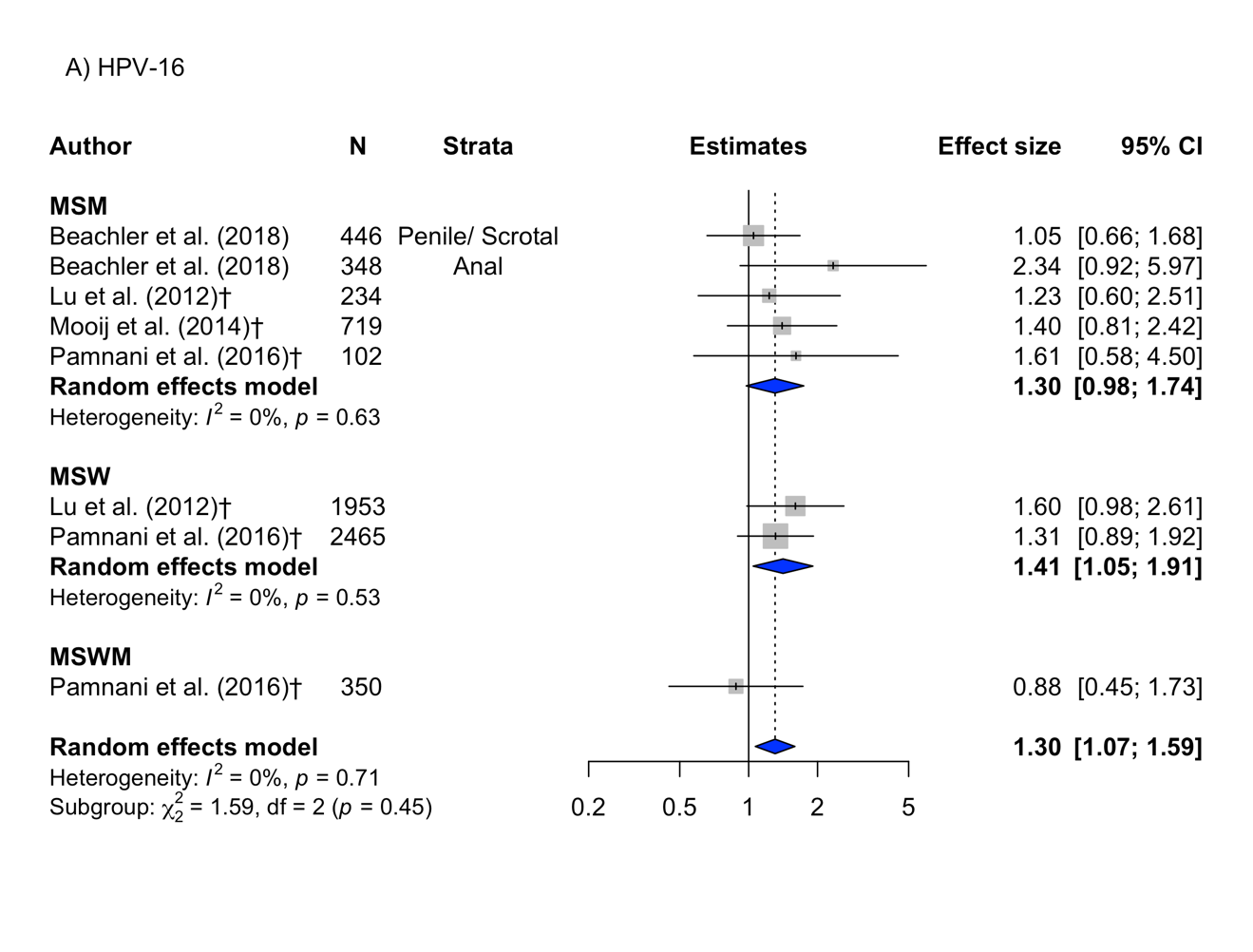
**

**
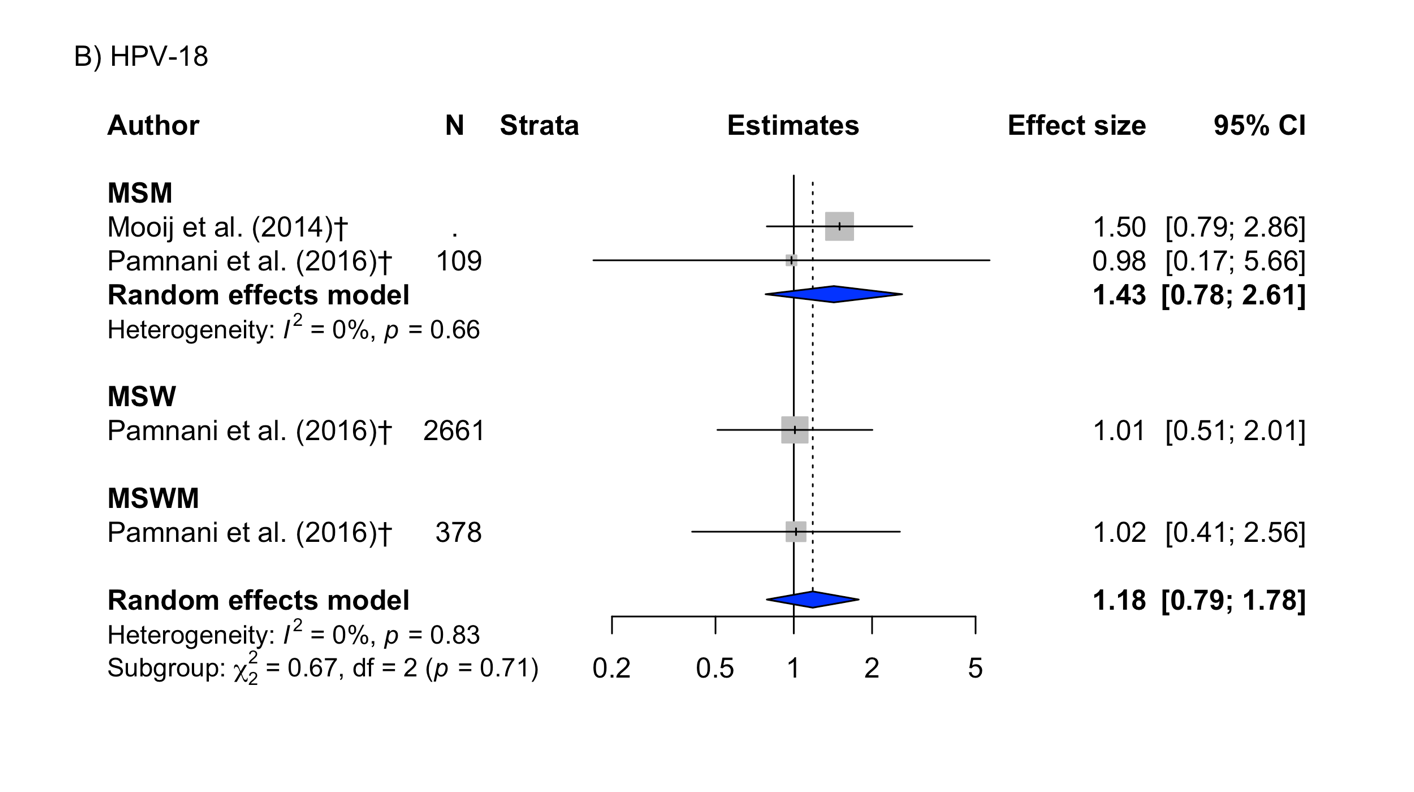
**

**Supplementary Figure 5.** Forest plot of the association between baseline HPV serostatus and type-specific HPV incident infection by sexual orientation among male for A) HPV-16 and B) HPV-18.

MSM=Men who have sex with men; MSW=Men who have sex with women; MSWM= Men who have sex with men and women; † indicates estimates that are adjusted for confounders; NA=not available.


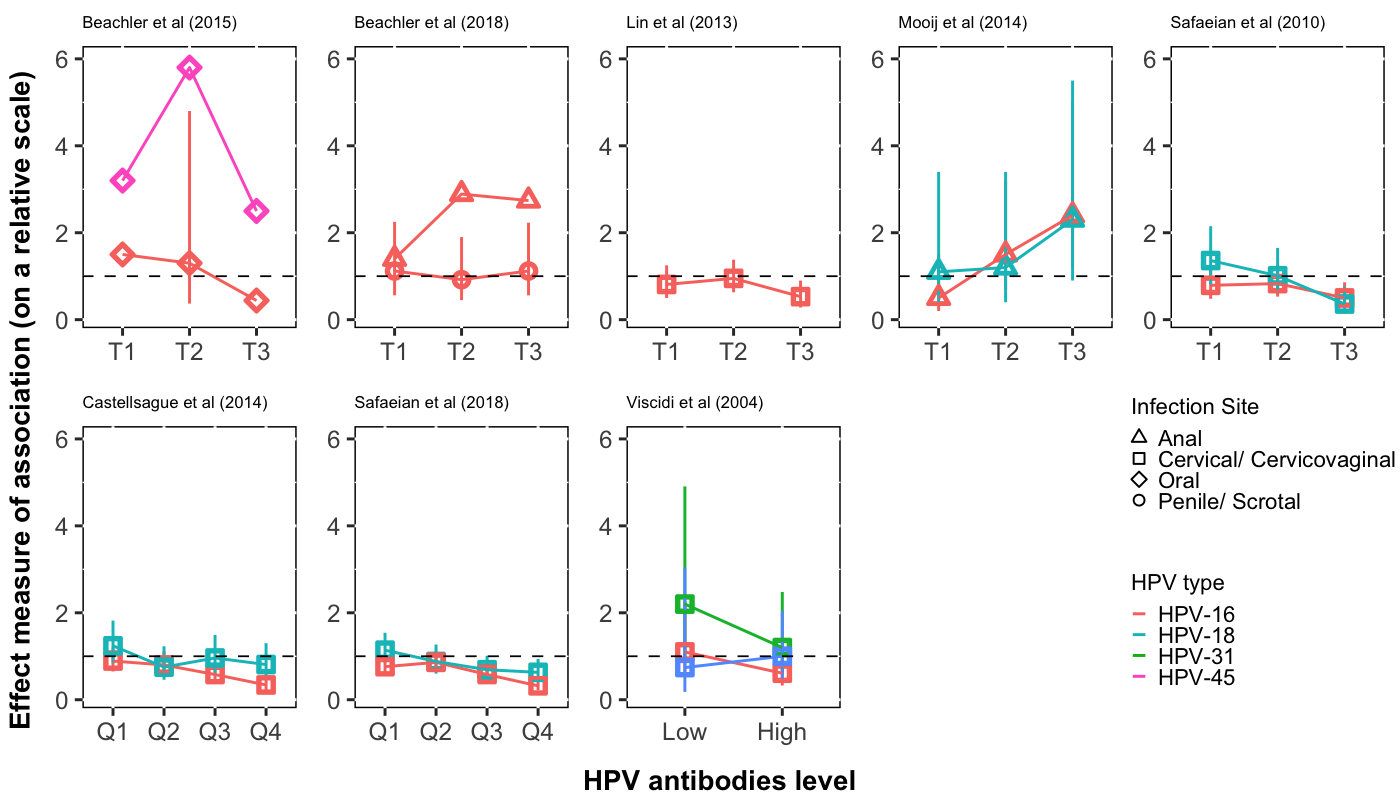


**Supplementary Figure 6.** Trends in association between baseline HPV serostatus and type-specific HPV incident infection by HPV antibody concentration level, stratified by publication**.**

1. **Females, HPV-16**


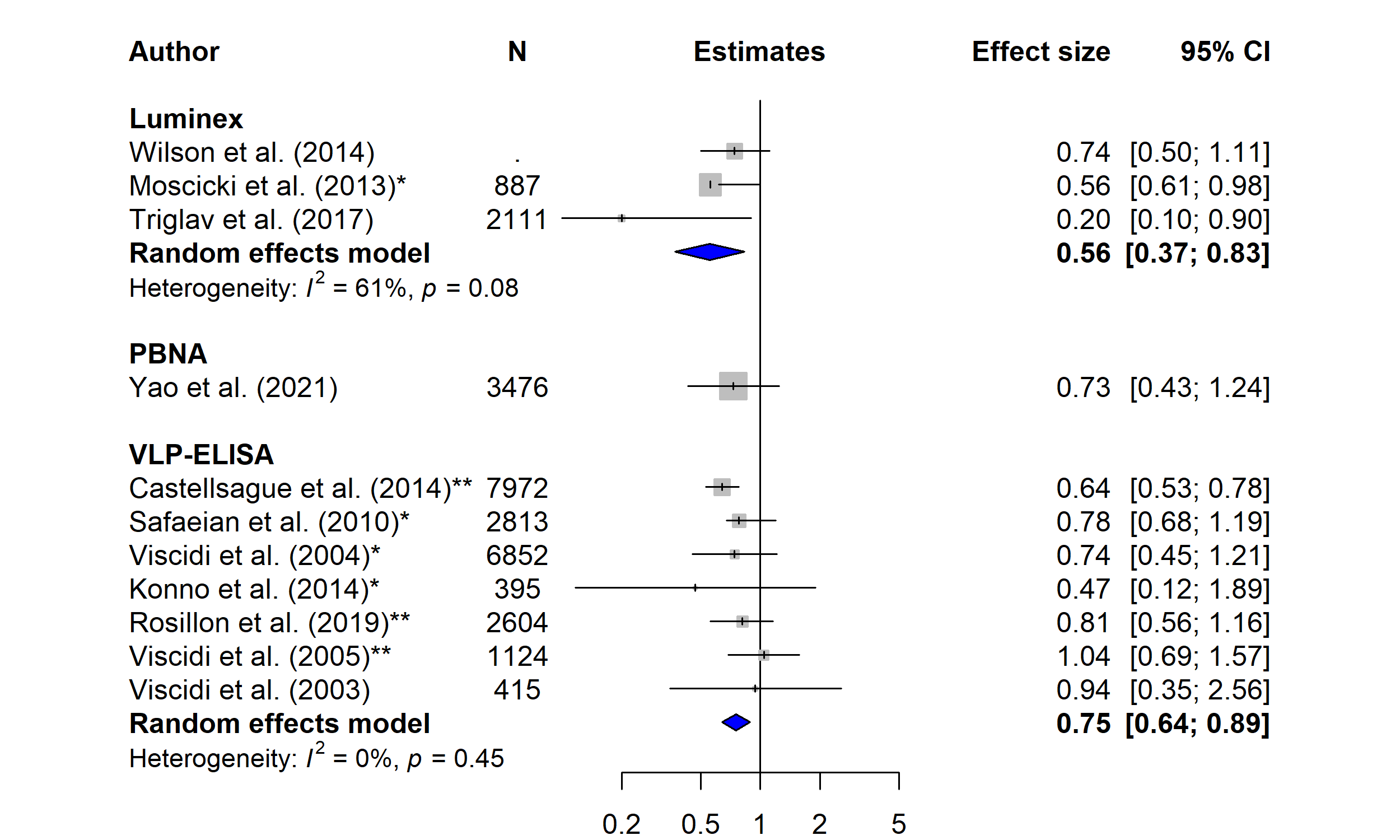


1. **Females, HPV-18**

**
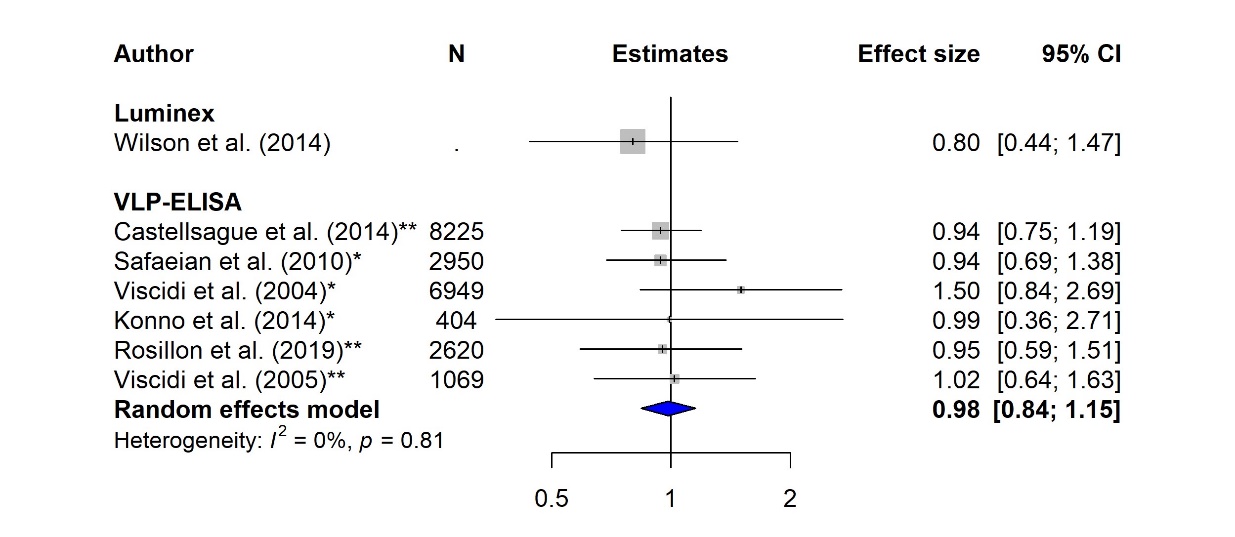
**

**Supplementary Figure 7.** Forest plot of the association between baseline HPV serostatus and type-specific HPV incident infection by serologic assay used among females for A) HPV-16 and B) HPV-18.

*indicates estimates that are self-calculated using the data presented in each publication; ** indicates estimates that are adjusted for confounders; NA=not available. Estimates from oral site were measured among male subjects.

**A) Males, HPV-16**

**
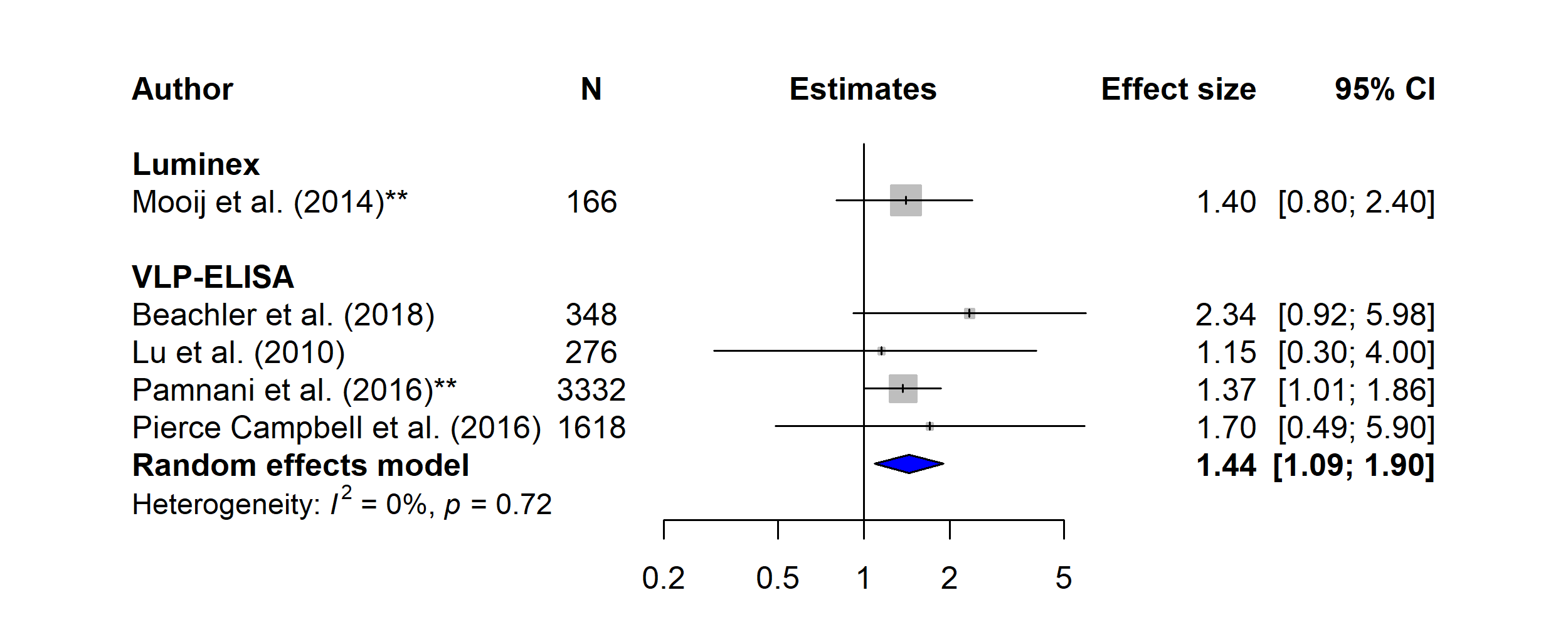
**

1. **Males, HPV-18**


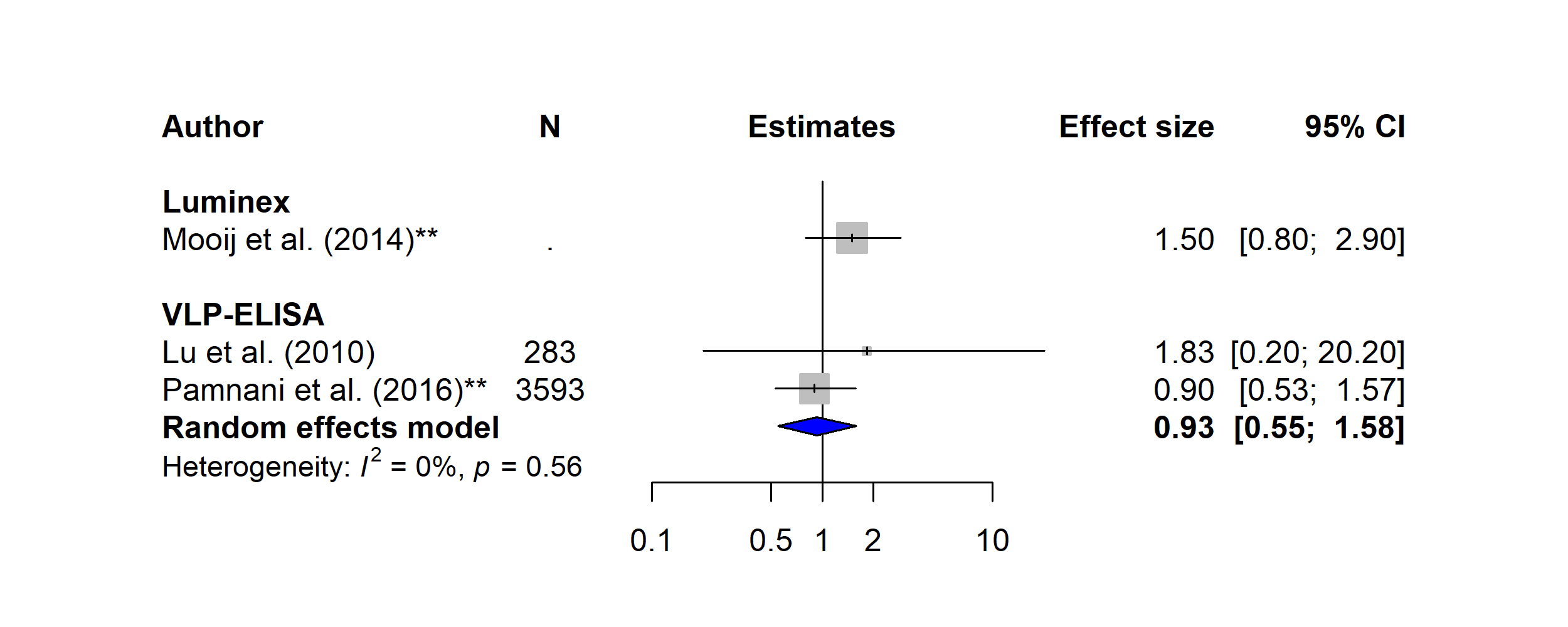


**Supplementary Figure 8.** Forest plot of the association between baseline HPV serostatus and type-specific HPV incident infection by serologic assay used among males for A) HPV-16 and B) HPV-18.

*indicates estimates that are self-calculated using the data presented in each publication; ** indicates estimates that are adjusted for confounders; NA=not available. Estimates from oral site were measured among male subjects.

**A) Females, HPV16**

**
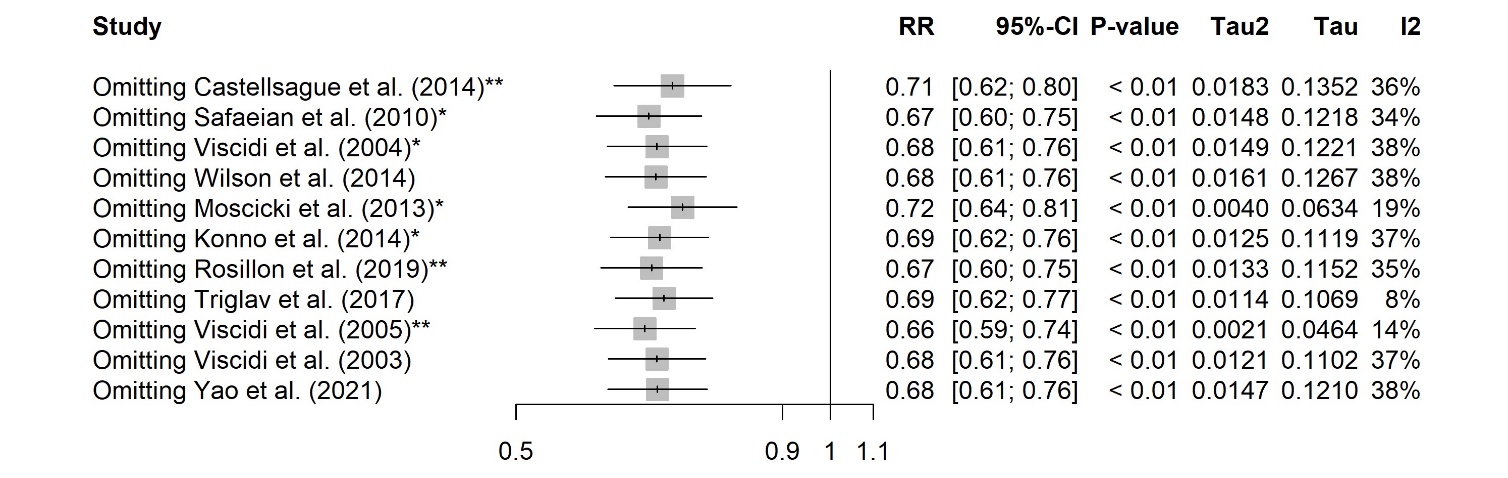
**

**B) Females, HPV-18**

**
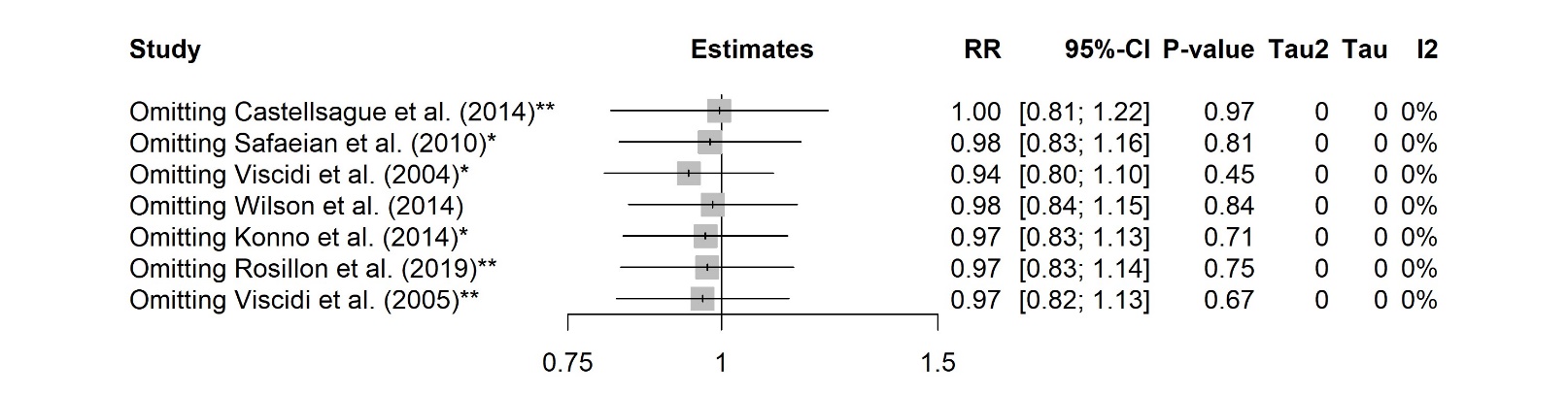
**

**C) Males, HPV-16**

**
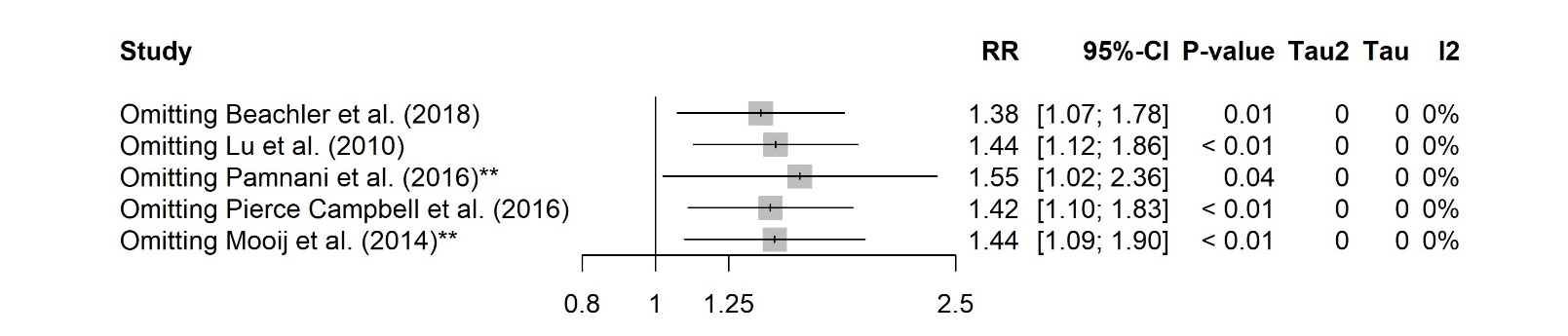
**

**D) Males, HPV-18**

**
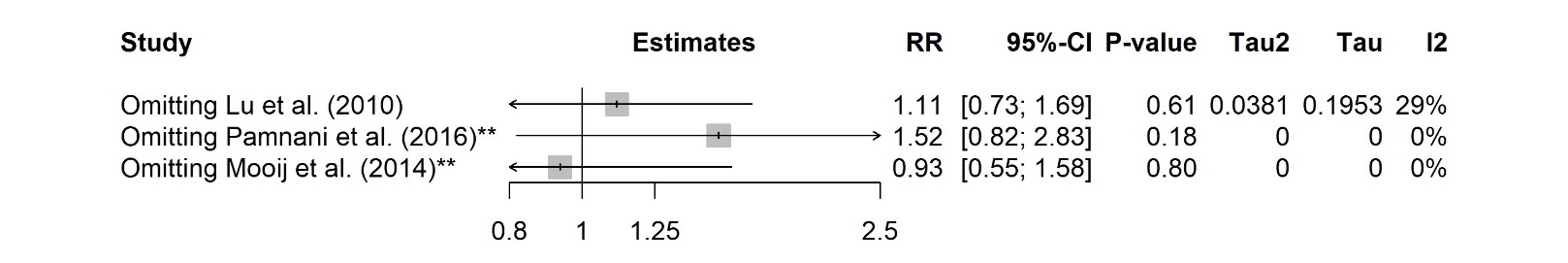
**

**Supplementary Figure 9.** Leave-one-out assessment of pooled associations between baseline HPV serostatus and type-specific HPV incident infection.

The above plot shows the pooled estimates omitting each effect sizeTheta-hat indicates the pooled estimates accompanied by the 95% CI. *indicates estimates that are self-calculated using provided data.

1. **HPV-16**

**
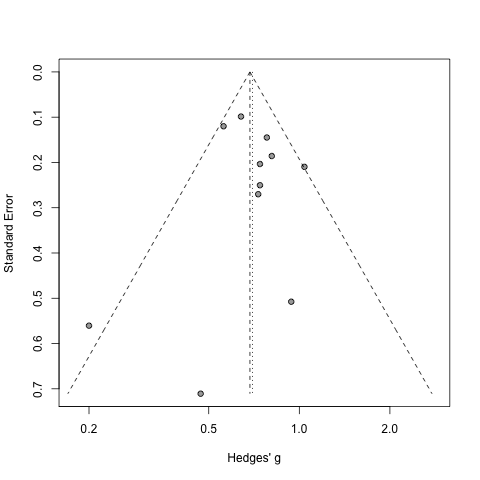

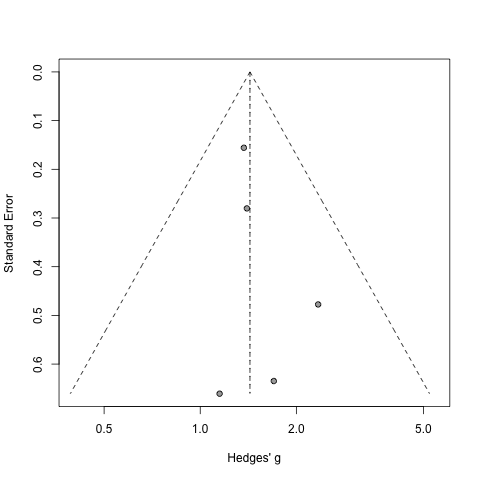
**

A1) Females A2) Males

1. **HPV-18**

**
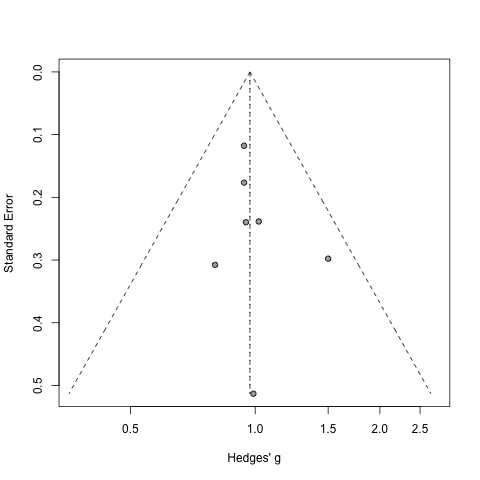

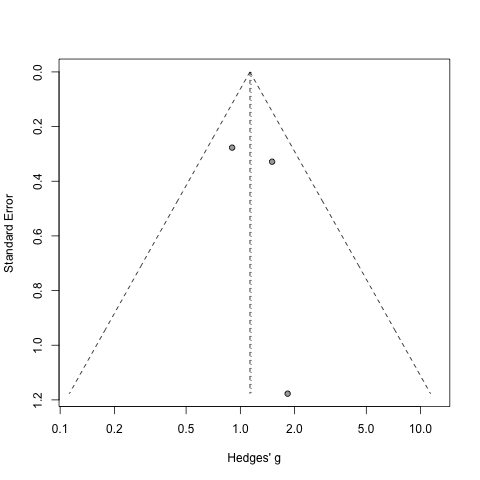
**

B1) Females B2) Males

**Supplementary Figure 10.** Funnel plot of the association between baseline HPV serostatus and type-specific HPV incident infection for A) HPV-16, B) HPV-18, by sex

**Supplementary Text 1. Notes on search strategy and statistical analysis**

Search strategy and selection criteria

We searched the Embase and MEDLINE databases for articles published up to October 25, 2020. We used key search terms related to four domains – HPV, study design, antibodies, and viral DNA– to capture prospective studies assessing an association between HPV serostatus at baseline (history of previous HPV infection) and subsequent type-specific HPV infection (HPV DNA detection at follow-up among DNA-negative participants at baseline; see supplementary materials). An additional domain was used to exclude cross-sectional and cost-effectiveness studies. The search was limited to articles published in English and, for MEDLINE, articles in French and Japanese were also considered.

Two reviewers (KY and KG) screened titles and abstracts of identified articles after removing duplicate records and resolving any discrepancy in the selection between the two reviewers. We excluded reviews, modelling studies, qualitative studies, and case-report studies, as well as studies that were not prospective in nature, studies without an assessment of HPV serostatus at baseline, and HPV DNA detection at baseline and follow-up. Full texts of all remaining relevant articles were then screened. We excluded publications that included HPV-DNA positive individuals at baseline (i.e., currently infected individuals), included HPV-vaccinated individuals, did not report or measure baseline HPV serostatus for past infection, did not report an estimate of the association between HPV serostatus at baseline and type-specific HPV incident infection at follow-up, or did not report any data allowing to self-calculate it. Finally, the references lists of all included publications were screened to identify potential additional relevant studies [1].

Data extraction

Data were retrieved and compiled in a standardized form by two reviewers (KY and KG) and any discrepancies were resolved by consensus. We extracted information on participants characteristics and HPV infection (i.e., sex, age, population type, HIV status, HPV type, infection site), study characteristics (i.e., country, study design, sample size, follow-up duration), and quality indicators (i.e., type of tests used for HPV serology and HPV DNA detection, variables adjusted for). We extracted the reported measures of association between serostatus of all HPV-type reported and incident infection. These include the incidence rate ratio (IRR), cumulative incidence ratio or risk ratio (RR), hazards ratio (HR), or the odds ratio (OR) and 95% confidence intervals (95%CI) or the data to derive RR or IRR (herein referred to as *self-calculated*) from reported counts and/or incidence rates (Supplementary materials).

Statistical analyses

We calculated the pooled measure of association (on the relative risk scale) and 95%CI using inverse variance weights and the DerSimonian-Laird random-effect method on the logarithmic scale [2]. To investigate how protective naturally-acquired antibodies are against subsequent HPV infection and maximize the number of estimates, we pooled any measures of association (IRR, RR, HR, OR) in our main meta-analysis. The I^2^ statistic was used to assess heterogeneity between study estimates [3]. To avoid including duplicated estimates on same participants, we only selected one estimate for each study population (e.g., HIM cohort study) for each HPV type, infection site, and HIV status. When several estimates were available from different studies on the same study population, we preferentially included estimates based on the largest sample size. When multiple types of measures of association were reported and/or derived for the same outcome within a publication (e.g., incidence rate ratio and risk ratio), we preferentially included estimates in the following order: IRR, HR, RR, and OR at last. We assumed that the OR was a reasonable approximation of the RR since all studies that only reported OR had an HPV cumulative incidence below 10% [4]. Finally, estimates that were adjusted for potential confounders were chosen over unadjusted estimates if available for the pooled analysis.

**Supplementary Text 2. Formulas used to calculate 95% CI for the self-calculated IRR and RR**

When the incidence rate ratio was self-calculated based on the data reported by the publication, or its 95% CI was not reported, 95% CI was calculated as follows:

- Calculate the natural log of the incidence rate ratio log(IRR)
- Calculate the standard error of the log(IRR) by the following:

$$SE(log \left( IRR \right))= \sqrt{\frac{1}{a}+\frac{1}{b}}$$

Where a and b correspond to the no. of incident cases as in the two by two table below:

| Baseline serostatus | No. of individuals with DNA detection at follow-up | Person-years contributed |
| --- | --- | --- |
| Seronegative | a | c |
| Seropositive | b | d |

- Calculate the lower and upper bounds of the confidence intervals on the natural log scale, and take the antilogarithm to obtain the upper and lower bounds of the confidence intervals:

$$95\% CI: [e^{\log\left( IRR \right)-[1.96\times SE\left( \log\left( IRR \right) \right)}, e^{\log\left( IRR \right)+[1.96\times SE\left( \log\left( IRR \right) \right)}]$$

When the cumulative incidence ratio was self-calculated based on the data reported by the publication, or its 95% CI was not reported, 95% CI was calculated as follows:

- Calculate Ln(RR)
- Calculate the standard error of the Ln(RR) by the following:

$$SE\left( Ln\left( RR \right) \right)= \sqrt{(\frac{n_{1}-x_{1}}{x_{1}})/ n_{1}+ (\frac{n_{2}-x_{2}}{x_{2}})/ n_{2}}$$

Where n corresponds to the no. of susceptible individuals at baseline and x, to the no. of non-cases as in the two by two table below:

| Baseline serostatus | No. of individuals without infection at follow-up | No. of individuals with infection at follow-up | Total no. of individuals included in the baseline |
| --- | --- | --- | --- |
| Seronegative | $x_{1}$ | $n_{1}-x_{1}$ | $n_{1}$ |
| Seropositive | $x_{2}$ | $n_{2}-x_{2}$ | $n_{2}$ |

- Calculate the lower and upper bounds of the confidence intervals on the natural log scale, and take the antilogarithm to obtain the upper and lower bounds of the confidence intervals:

$$95\% CI: [e^{\ln\left( RR \right)-[1.96\times SE\left( ln(RR) \right)}, e^{ln(RR)+[1.96\times SE(\ln\left( RR \right))}]$$

**Supplementary Text 3. Adapted Ottawa-Newcastle scale for the quality assessment**

**Selection** (max 6 stars)

**1) Representativeness of the sample** (max 1 star)

a) truly representative of the average target population *(All subjects selected from the population or random sampling methods used*.)*

b) somewhat representative of the average target population (*Non-random sampling methods may be used e.g. convenience sampling or unweighted RDS, but sample representative of target population*)*

c) selected group of users (*All selected participants are from a single population, which is not representative of the target population e.g. MSM selected from a sexual health clinic*)

d) no description of the sampling strategy

**2) Non-respondents** (max 1 star)

a) the response rate is satisfactory (>60%) and respondents and non-respondents are comparable**

b) the response rate is satisfactory (>60%) but the comparison between respondents and non-respondents is either not assessed and reported, or unsatisfactory*

c) the response rate is unsatisfactory (<60%) but respondents and non-respondents are comparable*

d) the response rate is unsatisfactory (<60%) and the comparison between respondents and non-respondents is either not assessed and reported, or unsatisfactory

**3) Assessment of exposure** (max 2 stars) ^a^

a) Detection of total HPV antibodies – Total ab test**

b) Detection of protective HPV antibodies - Neutralizing assay*

c) Information on the type of immunoassay used for antibodies detection is not available

**4) Demonstration that outcome of interest was not present at start of study** (max 1 star)

a) HPV-DNA positive individuals at baseline are excluded*

b) no

**Comparability** (max 1 star)

**1) Comparability of cohorts (HPV-seropositive vs seronegative groups) on the basis of the design or analysis** (max 1 star)

a) study controls (adjusts) for confounders*

b) study does not control (adjust) for confounders

**Outcome** (max 5 stars)

**1) Assessment of outcome** (max 3 stars)

a) HPV-DNA amplification with PGMY09/11 L1 PCR***

b) HPV-DNA amplification with SPF10 PCR**

c) HPV-DNA amplification with MY09/MY11 L1 PCR*

d) Information on primers used for HPV-DNA amplication not available

**2) Was follow-up long enough for outcomes to occur** (max 1 star)

a) yes - at least 6 months*

b) no - less than 6 months

**3) Adequacy of follow-up / lost to follow-up** (max 1 star)

a) complete follow up - all subjects accounted for *

b) subjects lost to follow up unlikely to introduce bias - small number lost (<10%), or those lost were comparable to those not lost *

c) low follow up rate (<90%) and no description of those lost

d) follow up rate and comparison between those lost and those not lost not reported

Notes:

1. when publications used more than one serological assay, a score for the highest-scored assay was applied
